# Toward understanding the brain tissue behavior due to preconditioning: an experimental study and RVE approach

**DOI:** 10.3389/fbioe.2024.1462148

**Published:** 2024-10-08

**Authors:** Ava Mazhari, Mehdi Shafieian

**Affiliations:** Department of Biomedical Engineering, Amirkabir University of Technology (Tehran Polytechnique), Tehran, Iran

**Keywords:** preconditioning, multiscale simulation, histological investigation, brain tissue microstructure, FEA, embedded element technique

## Abstract

Brain tissue under preconditioning, as a complex issue, refers to repeated loading-unloading cycles applied in mechanical testing protocols. In previous studies, only the mechanical behavior of the tissue under preconditioning was investigated; However, the link between macrostructural mechanical behavior and microstructural changes in brain tissue remains underexplored. This study aims to bridge this gap by investigating bovine brain tissue responses both before and after preconditioning. We employed a dual approach: experimental mechanical testing and computational modeling. Experimental tests were conducted to observe microstructural changes in mechanical behavior due to preconditioning, with a focus on axonal damage. Concurrently, we developed multiscale models using statistically representative volume elements (RVE) to simulate the tissue’s microstructural response. These RVEs, featuring randomly distributed axonal fibers within the extracellular matrix, provide a realistic depiction of the white matter microstructure. Our findings show that preconditioning induces significant changes in the mechanical properties of brain tissue and affects axonal integrity. The RVE models successfully captured localized stresses and facilitated the microscopic analysis of axonal injury mechanisms. These results underscore the importance of considering both macro and micro scales in understanding brain tissue behavior under mechanical loading. This comprehensive approach offers valuable insights into mechanotransduction processes and improves the analysis of microstructural phenomena in brain tissue.

## 1 Introduction

Brain tissue is among the most essential, intricate, and adaptable tissues in the human body, playing a pivotal role in both normal function and disease. Neurological conditions such as stroke, encephalitis, dementia, and epilepsy are recognized by the World Health Organization as major public health issues, with traumatic brain injuries affecting over 2 million people each year (Majdan et al., 2016). The complexity of brain tissue is evident in its composition, which includes neurons, glial cells, and the extracellular matrix (ECM) (Barros et al., 2011). The ECM is vital for brain development, maintenance, and involvement in disease mechanisms (Fawcett and Verhaagen, 2018). Glial cells—comprising astrocytes, oligodendrocytes, and microglia—along with axons, are essential for the central nervous system’s development, stability, and response to injury (Verkhratsky et al., 2016). Damage to axons can result in significant neurological impairments, compromising both the structural integrity of brain tissue and overall neurological function, often with serious consequences for a patient’s life (Johnson et al., 2013).

While traditional approaches in neuroscience have provided substantial insights into brain function through electrophysiological, biochemical, molecular, and genetic methods, recent research has highlighted the significant influence of mechanical forces on neuronal function and dysfunction, particularly in the context of traumatic brain or spinal cord injuries (Barnes et al., 2017; Goriely et al., 2015; Tyler, 2012; Li et al., 2011). The brain is located in the cranial cavity and is well-protected from mechanical stress under normal physiological conditions by the cerebrospinal fluid and meninges. However, abnormal loads can occur in pathological conditions such as traumatic brain injuries (Bruns Jr and Hauser, 2003; Mrozek et al., 2012; Urbanek and Frink, 2012), cancer and growing tumors (Pogoda et al., 2014), or brain swelling (Kolias et al., 2013), which may require surgical intervention. Therefore, conducting experimental mechanical characterization of brain tissue is necessary to better understand the mechanisms of injury.

Numerous *ex vivo* experiments have been conducted to characterize brain tissue. These experiments include shear (Arbogast and Margulies, 1998; Bilston et al., 2001; Darvish and Crandall, 2001; Hrapko et al., 2008; Rashid et al., 2013; Destrade et al., 2015; Budday et al., 2017), tensile (Budday et al., 2017; Miller and Chinzei, 2002; Velardi et al., 2006; Franceschini et al., 2006; Rashid et al., 2014; Eskandari et al., 2021; Jin et al., 2013), compression (Hrapko et al., 2008; Li et al., 2019; Eskandari et al., 2021; Miller and Chinzei, 1997; Cheng and Bilston, 2007; Laksari et al., 2012; Rashid et al., 2012; Li et al., 2019), and indentation (MacManus et al., 2020; Elkin et al., 2010; Li et al., 2019; Prevost et al., 2011; Lee et al., 2014; Budday et al., 2015; Samadi-Dooki et al., 2017; MacManus et al., 2018; Qian et al., 2018) tests. Different species, including humans (Menichetti et al., 2020; Sundaresh et al., 2022; Reiter et al., 2023; Jin et al., 2013; Budday et al., 2017; Franceschini et al., 2006; Shuck and Advani, 1972), monkeys (Galford and McElhaney, 1970; Metz et al., 1970), bovines (Bilston et al., 2001; Eskandari et al., 2021; Budday et al., 2015; Weickenmeier et al., 2016), porcines (Arbogast and Margulies, 1998; Thibault and Margulies, 1998; Velardi et al., 2006; Miller and Chinzei, 1997), and rodents (Koser et al., 2015; MacManus et al., 2018; Shulyakov et al., 2011; Antonovaite et al., 2021; Finan et al., 2012), have been used in these studies.

Despite the challenges related to brain tissue as an extremely soft and highly fragile tissue with heterogeneous behavior, diversity in species, and testing protocols, comparing the results of these mechanical tests is difficult. However, the results obtained from various experimental observations in many studies over the years agree on certain things, for instance, a strain-stiffening behavior, meaning that the stiffness increases with increasing strain (Budday et al., 2017). There exists a distinction in the structure and magnitude of tension and compression responses, known as tension-compression asymmetry (Jin et al., 2013; Budday et al., 2017; Miller and Chinzei, 2002; Rashid et al., 2014; Eskandari et al., 2021; Wang and Sarntinoranont, 2019). Moreover, there are notable variations in the characteristics of tension and compression within different regions of the brain, particularly between gray matter and white matter (Prange and Margulies, 2002; Jin et al., 2013; Budday et al., 2017; Velardi et al., 2006; Koser et al., 2015; Finan et al., 2012). Whether brain tissue is damaged under these deformations depends on our understanding of its mechanical properties.

Experimental methods have provided valuable insights, but they often fail to replicate the brain’s intricate *in vivo* mechanical environment. One of the key challenges in studying brain tissue is its extreme sensitivity and soft nature, which makes *in vivo* testing particularly difficult and potentially damaging. This limitation underscores the necessity for computational models, which can simulate tissue behavior under various loading conditions and offer a more accurate understanding of brain tissue’s mechanical responses in a controlled environment (Goriely et al., 2015). However, traditional computational models, such as those based on viscoelastic or porous media theories, often overlook the complex microstructural arrangements within the brain, particularly the role of axons. Axons are critical for maintaining the structural and functional integrity of neural networks, and their damage can lead to severe neurological deficits (Smith et al., 2013). Given this, our study focuses on developing a micromechanical model that specifically incorporates the arrangement and behavior of axons under repetitive cyclic loading. This focus on axonal microstructure is novel, as most existing models do not account for the detailed organization of axon fibers and their response to mechanical forces, especially in the case of preconditioning.

A highly complex issue raised by Fung is preconditioning, which involves repetitive loading-unloading cycles applied in test protocols, aiming to achieve a consistent and reproducible tissue response (Fung, 1993). Soft biological tissues typically undergo a pre-testing preparation to determine their material properties. Preconditioning may replicate the *in vivo* loading conditions in an *ex vivo* setting (Carew et al., 2000), that emphasizes the importance of repetitive cyclic loading applied in pre-testing stage of the experiments for soft tissues. In general, tissue preconditioning aims to achieve a robust tissue mechanical response, which leads to reducing the statistical variability in measurements. Early studies on soft tissue properties revealed that the tissue response followed a consistent loading and unloading path after three preconditioning cycles. Due to these advantages, preconditioning has become a standard protocol in many biological tissue tests, including the brain (Cheng et al., 2009; Prevost et al., 2011; Gefen and Margulies, 2004). Numerous researchers have investigated the stress-strain and stress-relaxation responses of biological tissues under various combinations of strain and strain rate. In such cases, typical protocols involve cyclic preconditioning, typically ranging from 3 to 10 cycles, applied at the specific strain and strain rate corresponding to the actual test conditions. This means that for studies involving testing at different combinations of strains and strain rates, the preconditioning protocols may differ for each condition. The effects of various preconditioning protocols have been examined in various tissues, including the aortic valve (Carew et al. (2000; 2004)), cardiac muscle (Pinto and Patitucci, 1980), ligament (Woo et al., 1982), tendon (Sverdlik and Lanir, 2002), and brain (Gefen et al., 2003).

A significant contribution of our study lies in the integration of detailed histological data with a micromechanics modeling framework to better understand the preconditioning phenomenon. While similar models have been developed in previous research, our approach advances the field by incorporating experimental data from histological analysis to capture microstructural changes in axon arrangements under cyclic loading. This particularization of the microstructure is essential, as it allows us to observe the specific alterations at the microscopic level that contribute to changes in the macroscopic mechanical behavior of brain tissue. This histological investigation represents a key advance, providing novel insights into the brain’s microstructural response to preconditioning, which has not been thoroughly explored in prior studies. Furthermore, our micromechanics framework offers a refined and more accurate representation of these changes, linking microstructural behavior to overall tissue mechanics. By focusing on both the experimental and modeling aspects, our study bridges a critical gap in understanding the interplay between brain tissue microstructure and its mechanical properties during preconditioning cycles.

In the case of brain tissue, previous experimental studies have shown that preconditioning is not linked to permanent tissue damage. However, relatively reversible alterations in tissue state, such as the removal of interstitial fluid or intra-cellular interactions, also lead to making brain tissue soften (Budday et al., 2020). The microstructure of the brain plays a critical role in determining its macroscopic behavior. However, previous experiments have provided some insights, without capturing the subtle microstructural changes that occur during preconditioning. Therefore, the exact influence of the microstructure and its changes due to preconditioning still needs to be fully understood.

Our model aims to bridge this gap by focusing on the microstructural arrangement of axon fibers within the brain, allowing us to observe how these structures respond to preconditioning cycles. This approach is particularly important because changes at the microstructural level can significantly influence the tissue’s overall mechanical behavior (Geers et al., 2010; Holzapfel and Fereidoonnezhad, 2017). By incorporating detailed microstructural data into our computational model, we aim to provide a more accurate representation of brain tissue mechanics under preconditioning.

To better understand the relationship between microstructure and mechanical behavior, the objective of this study is to develop a model with the micromechanical finite element modeling approach based on the experimental data from brain tissue to observe microstructure changes and its effect on the macroscale under preconditioning. Our approach provides a novel perspective by explicitly incorporating the arrangement of axons and their microstructural changes under cyclic loading. This allows us to investigate the effects of preconditioning on brain tissue mechanics in a way that has not been studied before. First, we provide an overview of soft tissue’s general mechanical principles and modeling’s mechanical foundations employed in this study. Subsequently, using an experimental study, we used data from mechanical testing and analysis of histological images to create a micromechanical model that enabled us to optimize our model’s material parameters. In particular, we model the quasi-static microstructural changes in the arrangements of axon fibers under preconditioning by using the embedded element technique in the representative volume elements (RVE) models. A sensitivity analysis was conducted to determine the optimal edge length and mesh size for both models. The hyperelastic material properties of the ECM and axonal fibers were characterized for both models using a multi-objective evolutionary optimization procedure, ensuring that the homogenized stress responses closely matched the experimental curves. The validity of the optimally characterized models was evaluated by comparing the predicted homogenized responses of the white matter structures with the experimental data presented in this study. Finally, we conclude by discussing the key findings of our research.

## 2 Theoretical preliminaries

Computational modeling provides a valuable tool for analyzing and predicting the behavior of human brain tissue under various loading conditions. However, the accuracy of numerical predictions relies on the selection of appropriate constitutive models. In the following section, we will briefly discuss the mathematical formulations used to capture the characteristics of brain tissue behavior. The complexity of tissue response varies depending on the loading conditions; accordingly, different modeling approaches are required. The same material may necessitate different constitutive relations based on the specific application.

In this study, the consideration of viscoelasticity was omitted due to the assumption that the effect of viscosity is negligible under the quasi-static loading conditions. Although preconditioning can involve viscous effects, studies suggest that these effects can also be modeled within a hyperelastic framework, particularly under quasi-static conditions where the material’s response evolves path-dependently (Holzapfel and Ogden, 2009; Ehret and Itskov, 2007; Mihai and Goriely, 2017). Similarly, the simulation parameters of slow processes should be calibrated using the preconditioned tissue response, while the parameters of fast processes should usually be based on the non-preconditioned response, ideally probed at high rates (Budday et al., 2020). Hyperelasticity is therefore suitable for capturing the strain-stiffening and tension-compression asymmetry observed in brain tissue during large deformations, phenomena that are better explained by intrinsic nonlinear elastic properties rather than viscous effects. We will represent constitutive relations of increasing complexity to capture the time-independent behaviors of brain tissue. Given the high compliance of brain tissue and its significant nonlinearity, even at strains as low as 1%, we focus exclusively on constitutive models based on nonlinear field theory of mechanics.

### 2.1 Hyperelasticity

Initially, our focus lies on examining the response of brain tissue that is independent of time, disregarding any contributions from viscosity or porosity. The critical characteristics observed in this time-independent state are nonlinearity and an asymmetry between compression and tension response. We propose a strain-energy function, ψ(F) , defined per unit reference volume and dependent only on the deformation gradient F . Prior studies have suggested fiber-reinforced material models for brain tissue, where the strain energy depends not only on the deformation gradient F but also on the direction of the fibers (Cloots et al., 2011; Feng et al., 2017; Ning et al., 2006; Arbogast and Margulies, 1999; Giordano and Kleiven, 2014; Garcia-Gonzalez et al., 2018). Studies have demonstrated that white matter exhibits characteristics of being a heterogeneous and anisotropic material (Jin et al., 2013). Additionally, it has been documented that brain tissue demonstrates incompressible hyperelastic behavior under large deformations (Libertiaux et al., 2011; MacManus et al., 2017; Rashid et al., 2012; Takhounts et al., 2003).

Various phenomenological, isotropic strain-energy functions have been suggested to describe brain tissue’s constitutive behavior (Budday et al., 2017; de Rooij and Kuhl 2016; Kaster et al., 2011; MacManus et al., 2018; Rashid et al., 2012). Among these material models, the one-term Ogden model proved to be successful in describing the approximate mechanical behavior of the tissue under different loading modes (Mihai et al., 2015; Budday et al., 2017), so in this study, we specifically consider one term Ogden model with the strain-energy function as (Ogden, 1972; de Rooij and Kuhl, 2016):

$$\psi =\frac{2\mu }{\alpha^{2}}\left(\lambda_{1}^{\alpha }+\lambda_{2}^{\alpha }+\lambda_{3}^{\alpha }-3\right)+\frac{1}{D}{\left(J-1\right)}^{2}$$ (1)

Here in Equation 1, the notation $\lambda_{i}$ for i = 1,2,3 represents the principal stretches, μ denotes the shear modulus, D is the material constant associated with the bulk modulus. The constitutive parameter α represents the nonlinear characteristics of the tissue that are sensitive to the magnitude of the strain, and J represents the determinant of the deformation gradient tensor.

### 2.2 Embedded element technique

The embedded element technique involves the integration of a reinforcement mesh of finite elements within a host mesh, allowing for the modeling of complex interactions between different components (Phillips and Zienkiewicz, 1976). This technique involved superimposing the guest domain (axons) onto the host domain (extracellular matrix), creating separate grids for each domain (Dassault Systèmes, 2021). By imposing a kinematic bond using shape functions, a strong coupling between the reinforcement and host meshes is achieved. This coupling allows for independent generation and analysis of the host and reinforcement mesh (Phillips and Zienkiewicz, 1976; Elwi and Hrudey, 1991; Garimella et al., 2019). As discussed in previous studies, embedded reinforcement models do not reproduce the discontinuity in the strain field (Goudarzi and Simone, 2019). These models usually indirectly calculate the displacement of an inclusion by using the relative displacement between the inclusion and the matrix as an additional field.

This technique works by constraining the translational degrees of freedom (DOFs) of the embedded nodes to align with the interpolated DOFs of the surrounding host element. Implementing the embedded element method to generate a heterogeneous representative volume element (RVE) offers several key benefits by reducing computational costs. The guest domain encompasses two material properties—one for the fiber and another for the matrix. Since the fibers are embedded within the matrix, the matrix’s material properties are superimposed onto those of the fiber, creating a combined material model within a single domain. The overall strain energy density for this domain can be represented as follows:

$$\psi_{\mathrm{Total}}\left(\lambda_{1},\lambda_{2},\lambda_{3}\right)=\psi_{f}\left(\lambda_{1}^{\prime },\lambda_{2}^{\prime },\lambda_{3}^{\prime }\right)+\psi_{m}\left(\lambda_{1}^{\prime \prime },\lambda_{2}^{\prime \prime },\lambda_{3}^{\prime \prime }\right)$$ (2)

Here, $\lambda_{i}=1,2,3$ are the principal stretches, and $\psi_{f}$ and $\psi_{m}$ are the strain energy densities of the guest and host domains, respectively. This analysis considers two distinct material models for axonal fibers and ECM, each with independent material constants. Because the DOFs of corresponding nodes in the guest and host domains are fully coupled in the embedded element method, the principal stretches are equal. By substituting the Ogden material model into Equation 2, and considering incompressibility for the fiber and extracellular matrix components (J=1) , the strain-energy density function can be reformulated for the fibers and the matrix as separate equations, denoted as Equations 3, 4, respectively.

$$\psi_{f}=\frac{2\mu_{f}}{\alpha_{f}^{2}}\left(\lambda_{1}^{\alpha_{f}}+\lambda_{2}^{\alpha_{f}}+\lambda_{3}^{\alpha_{f}}-3\right)$$ (3)

$$\psi_{m}=\frac{2\mu_{m}}{\alpha_{m}^{2}}\left(\lambda_{1}^{\alpha_{m}}+\lambda_{2}^{\alpha_{m}}+\lambda_{3}^{\alpha_{m}}-3\right)$$ (4)

Studies have demonstrated that the material constant “ α ” is not influenced by the loading direction (Meaney, 2003). As a result, assuming that “ α ” is equal for both the fibers and the matrix $\left(\alpha =\alpha_{f}=\alpha_{m}\right)$ , only three independent material constants, namely $\mu_{f},\mu_{m}$ and α would be necessary to fully describe the hyperelastic properties of the constituents of white matter (Yousefsani et al., 2018a).

To characterize the kinematics of the embedded element technique, as described in previous studies (Dalbosco et al., 2021), we have to consider a continuum body denoted as $\Omega_{0}$ , which undergoes quasi-static deformation to Ω (where $\Omega_{0},\Omega \subset R^{3}$ ). Points $X\in \Omega_{0}$ and x∈Ω represent the positions of a particle in the reference and current configuration respectively. The deformation of the body occurs as a result of the prescribed displacements represented by the field $\bar{U}:\partial \Omega_{0}^{u}\to R^{3}$ on the portion $\partial \Omega_{0}^{u}$ of the boundary $\partial \Omega_{0}$ . This displacement field, in combination with the body force field $\bar{B}:\partial \Omega_{0}\to R^{3}$ and the surface traction field $\bar{T}:\partial \Omega_{0}^{\sigma }\to R^{3}$ prescribed on the portion $\partial \Omega_{0}^{\sigma }$ of the boundary $\partial \Omega_{0}$ , contributes to the deformation process.

$$\partial \Omega_{0}^{u}\cup \partial \Omega_{0}^{\sigma }=\partial \Omega_{0},\;\partial \Omega_{0}^{u}\cap \partial \Omega_{0}^{\sigma }=Ø$$ (5)

Equation 5 indicates that the prescribed displacement and surface traction are defined on separate parts of the boundary $\partial \Omega_{0}$ . To properly formulate the boundary value problem (BVP), we begin by presenting the strong form of the problem. The governing differential equations are derived from the balance of linear momentum in the reference configuration, which, for a quasi-static case, can be expressed as Equation 6:

$$DivP+B=0,\;\text{in }\Omega_{0}$$ (6)

where P is the First Piola-Kirchhoff stress tensor, B is the body force per unit reference volume, and $\Omega_{0}$ is the reference configuration. The corresponding boundary conditions are:

$$u=u_{0},\;\text{on }\partial \Omega_{u}$$ (7)

$$P\cdot N=\bar{T},\;\text{on }\partial \Omega_{\sigma }$$ (8)

Here in Equations 7, 8, u is the displacement field, $u_{0}$ is the prescribed displacement on the boundary $\partial \Omega_{u}$ , N is the outward unit normal vector on the boundary, and $\bar{T}$ is the prescribed traction on the boundary $\partial \Omega_{\sigma }$ . With the strong form of the boundary value problem posed, we can now derive its weak form using the principle of virtual work. By integrating the equation over the reference configuration and applying the divergence theorem, we arrive at the following expression:

$$\begin{array}{c} F\left(u,\delta u\right)=\int_{\Omega_{0}}\left[P:Grad\delta u-\left(B-\rho_{0}\ddot{u}\right).\delta u\right]dV-\int_{\Omega_{0}^{\sigma }}\bar{T}\delta udS=0 \end{array}$$ (9)

The virtual displacement field, denoted as δu (defined on the reference configuration), satisfies the condition δu=0 on the part of the boundary surface $\Omega_{0}^{u}$ . The surface traction, represented by $\bar{T}$ , applies to the portion $\partial \Omega_{0}^{u}\subset \partial \Omega_{0}$ .

In Equation 9, the first piola-Kirchhoff stress tensor denotes as P and B are the first Piola-Kirchhoff stress tensor and the reference body force. This choice is consistent with the use of the reference configuration in defining the virtual work principle. B is the reference body force. Assuming quasi-static deformation, dynamical quantities such as $\rho_{0}\ddot{u}$ , the inertia force per unit reference point volume has to be negligible [see Ref (Holzapfel, 2002) for details]. So we can rewrite Equation 9 as:

$$\begin{array}{c} F\left(u,\delta u\right)=\int_{\Omega_{0}}\left[P:Grad\delta u-B.\delta u\right]dV-\int_{\Omega_{0}^{\sigma }}\bar{T}\delta udS=0 \end{array}$$ (10)

Assuming the no-slip boundary condition between the fibers and the matrix, the deformation gradient F is continuous over the boundary surface $\partial \Omega_{0}$ . We have specify the initial volume of the embedded fibers by $V_{0}^{f_{i}}$ where $V_{0}^{f_{\mathrm{Total}}}=\sum_{i=1}^{N_{f_{\mathrm{Total}}}}V_{0}^{f_{i}}$ represents the total volume of fibers.

The additional superimposed volume at the overlapping regions of the fiber and host domains increases the mechanical stiffness of the model, leading to an overestimation of stiffness due to the use of the embedded element technique. Based on the hyperelastic behavior of the matrix and the fibers, we could decompose the internal strain energy into two parts; $\psi_{m}$ for the matrix m and $\psi_{f_{\mathrm{Total}}}$ for the fibers $N_{f_{\mathrm{Total}}}$ .

Previous studies have been presented the correction of the addressed issue by subtracting strain energy density functions (SEDF) of the matrix from the fibers as shown in Equation 11 (Yousefsani et al., 2018b), which resolves the well-known issue of volume redundancy in models with embedded reinforcement, ensuring that the model accurately reflects the material’s true mechanical properties [discussed by (Garimella et al., 2019)]:

$$\psi_{c}\left(F\right)=\psi_{f_{i}}\left(F\right)-\psi_{m}\left(F\right)$$ (11)

where the $\psi_{c}\left(F\right)$ is the corrected strain energy function for embedded region, the $f_{i}$ and m sub-indices are in association with fibers and matrix, respectively. Subsequently, the principle of virtual work can be written as:

$$\begin{array}{c} \int_{\Omega_{0}}P_{m}:Grad\delta u+\overset{N_{f_{\mathrm{Total}}}}{\underset{i=1}{\sum }}\int_{\Omega_{0}}P_{c_{i}}:Grad\delta u-\int_{\Omega_{0}}\bar{B}.\delta udV-\int_{\Omega_{0}^{\sigma }}\bar{T}\delta udS=0 \end{array}$$ (12)

In Equation 12, $P_{m}$ and $P_{c_{i}}$ are the first Piola-Kirchhoff stress tensors of the matrix m and of the correction of the $i^{th}$ fiber $f_{i}$ , respectively and Gradδu represents the first variant of deformation gradient F .

It is important to note that the First Piola-Kirchhoff stress tensor P is employed throughout the formulation to maintain consistency in describing stresses in the reference configuration, which is essential when applying the principle of virtual work in the context of finite element analysis. Moreover, P facilitates the balance of linear momentum in the reference configuration, which is often convenient for the type of analysis presented in this study. Specifically, the relationship between the deformation gradient F and the stress tensors can be described as shown in Equation 13:

P=F⋅S (13)

where S is the Second Piola-Kirchhoff stress tensor, and F is the deformation gradient. This relation shows how the First Piola-Kirchhoff stress tensor P is directly connected to S , which is derived from the strain energy function. The use of P is thus justified as it enables the formulation to work with stress measures directly connected to the physical forces experienced in the current configuration while remaining in the reference configuration. This simplifies the formulation when large strains are involved, as P is consistent with both the deformation gradient and the boundary conditions in the weak form (as seen in Equations 9, 10, 12).

Supplementary Appendix S1 presents a finite element method (FEM) implementation designed for finite strain simulations.

The correction method is based on Ogden hyperelastic material formulation, discussed in Ref. (Yousefsani et al., 2018b) and the subtraction of shear modulus of matrix and fibers may be replace the corrected modulus for the embedded region as expressed as below:

$$\mu_{f}^{*}=\mu_{f}-\mu_{m}$$ (14)

The adjusted stiffness of the superimposed fibers, denoted as $\mu_{f}^{*}$ , is determined based on the fiber stiffness $\mu_{f}$ and the matrix stiffness $\mu_{m}$ .

### 2.3 Multiscale homogenization

Over the past decades, there has been significant progress in adopting multiscale theories to establish a connection between the macroscopic behavior of materials and the physical phenomena occurring at smaller scales, particularly in computational mechanics. In solid mechanics, notable contributions by Hill (1963); Hill (1965a); Hill (1965b); Hill (1972) Mandel (1971), among others, have provided a robust framework for estimating the macroscopic mechanical response of heterogeneous materials.

More recently, These theories have focused on the Representative Volume Element (RVE) concept, which enables the calculation of macro-scale stresses and strains by averaging their micro-scale counterparts within the RVE. The RVE is typically treated as a continuum, although discrete interactions can also be considered. The practical application of RVE-based theories relies on computational homogenization techniques, often utilizing finite element methods.

#### 2.3.1 Periodic boundary condition

**FIGURE 1:**
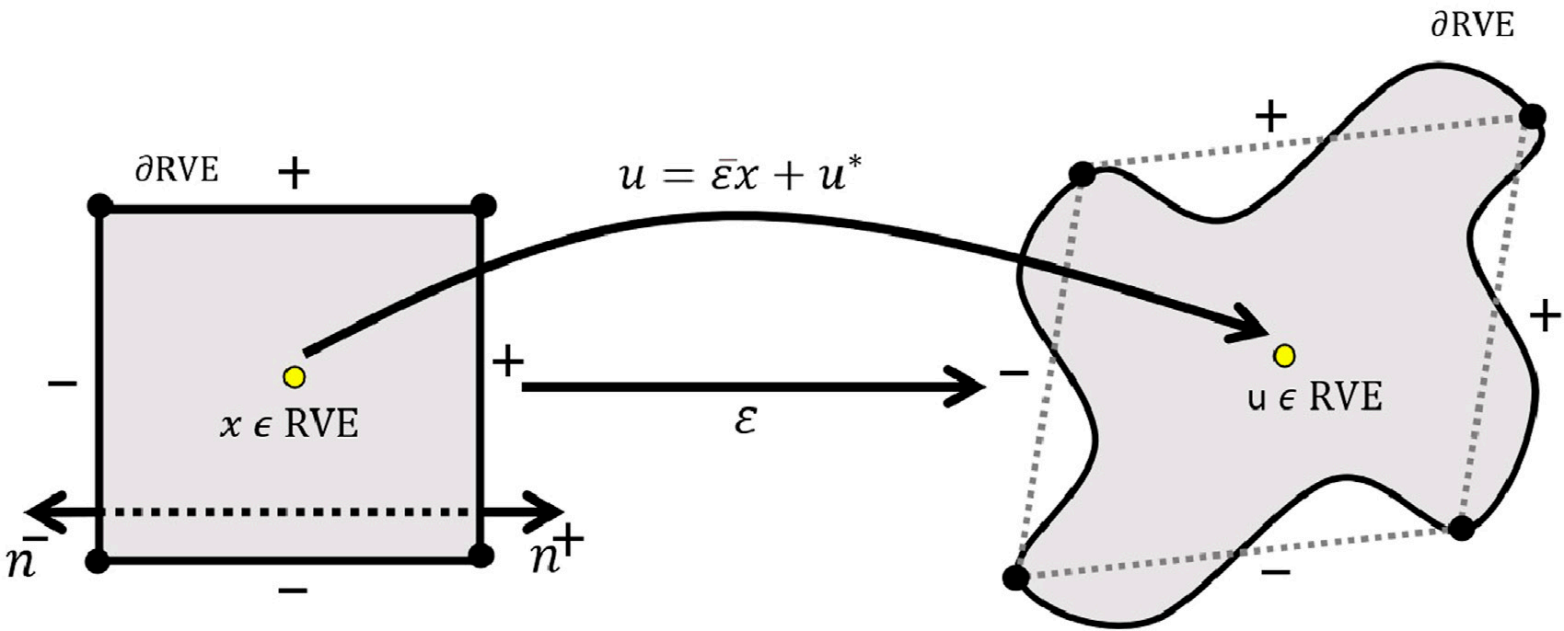
Application of periodic boundary conditions (PBC) on the boundaries of the representative volume element (RVE) in the microsampling domain.

To complete the mechanical boundary value problem of a network RVE $\left(\Omega_{\mathrm{RV E}}\subset R^{3}\right)$ , along with its constituents corresponding constitutive and structural models, it is necessary to specify appropriate boundary conditions. Previous studies have shown that adopting the periodic boundary condition, rather than the uniform tensile or linear displacement boundary condition, leads to more accurate approximations of the effective mechanical properties of composites (Bouaoune et al., 2016; Hazanov and Huet, 1994; Hori and Nemat-Nasser, 1999). Therefore, we will use the periodic boundary condition in this study to ensure more reliable results. In the case of a periodic RVE, the boundaries $\partial \Omega_{\mathrm{RV E}}$ of the RVE can be divided into two opposing parts: $\partial \Omega_{\mathrm{RV E}}^{+}$ and $\partial \Omega_{\mathrm{RV E}}^{-}$ (Figure 1). This division is indicated in Equation 15:

$$\begin{array}{c} \partial \Omega_{\mathrm{RV E}}^{+}\cup \partial \Omega_{\mathrm{RV E}}^{-}=\partial \Omega_{\mathrm{RV E}}\\ \partial \Omega_{\mathrm{RV E}}^{+}\cap \partial \Omega_{\mathrm{RV E}}^{-}=Ø. \end{array}$$ (15)

For each point $x^{+}$ on $\partial \Omega_{\mathrm{RV E}}^{+}$ , a unique corresponding point $x^{-}$ on $\partial \Omega_{\mathrm{RV E}}^{-}$ exists, and the normal vectors at these boundaries satisfy $n^{-}={-n}^{+}$ . It is essential to ensure that identical meshes are used on the opposite surfaces of the RVE to guarantee the convenient and efficient imposition of periodic boundary conditions. The displacement field u for the periodic RVE of composites, subjected to a macroscopic strain u , can be expressed as (Suquet, 1987):

$$u_{i}\left(x_{1},x_{2},x_{3}\right)=u_{i}^{0}+u^{*}\left(x_{1},x_{2},x_{3}\right),\;u_{i}^{0}=\varepsilon_{ij}^{0}x_{j}$$ (16)

The term $\;u_{i}^{0}=\varepsilon_{ij}^{0}x_{j}$ represents the linear displacement field within the RVE, which corresponds to the applied macroscopic strain assuming a homogeneous material. This linear field is crucial for ensuring that the boundary conditions imposed are consistent with the overall deformation of the RVE. The second term, $u^{*}\left(x_{1},x_{2},x_{3}\right)$ , is the periodic part of the displacement field and accounts for the modifications to the linear displacement caused by the heterogeneous structure of the composites.

In the context of hyperelastic materials, the linear displacement field $\;u_{i}^{0}$ serves to enforce boundary conditions necessary for maintaining the periodicity of the RVE. While hyperelasticity involves complex, non-linear material behavior, the periodic boundary conditions focus on the overall deformation applied to the RVE and the need to maintain continuity across its boundaries. Therefore, the linear displacement field is not a reflection of the material’s constitutive response but rather a tool to ensure the proper application of boundary conditions.

However, the displacement field expressed in Equation 16 cannot be directly applied to the boundaries of an RVE because the periodic part $u^{*}\left(x_{1},x_{2},x_{3}\right)$ is generally unknown. For any RVE of composites, its boundary surfaces always appear in parallel pairs. The displacements on a pair of parallel opposite boundary surfaces can be written as Equation 17:

$$u_{i}^{k+}=\varepsilon_{ij}^{0}x_{j}^{k+}+u_{i}^{*},\;\;u_{i}^{k-}=\varepsilon_{ij}^{0}x_{j}^{k-}+u_{i}^{*}$$ (17)

The indices k+ and k− refer to the $k^{th}$ pair of two opposing parallel boundary surfaces within an RVE of composites. It is important to note that the values of $u_{i}^{*}$ are identical at these parallel boundaries due to their periodic nature. Therefore, the distinction between the two equations mentioned above can be expressed as follows:

$$u_{i}^{k+}-u_{i}^{k-}=\varepsilon_{ij}^{0}\left(x_{j}^{k+}-x_{j}^{k-}\right)=\varepsilon_{ij}^{0}\Delta x_{j}^{k}$$ (18)

Since the term $x_{j}^{+}-x_{j}^{-}$ remains constant for every pair of nodes located on the parallel boundary surfaces, edges, and corner vertices within the RVE of composites, given a specific macroscopic strain $\varepsilon_{ij}$ , the right-hand side of Equation 18 also becomes constant. This allows for easy implementation of the equations as a nodal displacement constraint in finite element analysis. It is important to note that Equation 18 represents a particular type of displacement boundary condition, where the focus is on specifying the differences in displacement between two opposing boundaries rather than providing known values for boundary displacements. Applying Equation 18 ensures the continuity of the displacement field. Consequently, it is necessary to establish traction continuity conditions, which are denoted by the following expressions:

$$t_{i}^{+}=-t_{i}^{-}\;\;with\;\;t_{i}=\sigma_{ij}^{0}n_{j}$$ (19)

in the given context (Equation 19), the normal vectors at these boundaries satisfy the condition $n^{-}={-n}^{+}$ . This implies that the normal vectors on the boundary $\partial \Omega_{\mathrm{RV E}}$ for composites adhere to a periodic boundary condition, which can be expressed as follows:

$$u_{i}^{+}-u_{i}^{-}=\varepsilon_{ij}\left(x_{j}^{+}-x_{j}^{-}\right)=\Delta u_{i},\;\;t_{i}^{+}=-t_{j}^{-}$$ (20)

Equation 20 provides the uniqueness of the solution, making it unnecessary to explicitly apply the latter boundary condition in the finite element analysis. It is important to note that because displacement boundary conditions are not specified for any particular point, translational rigid body motions of the RVE of composites are allowed. The details of numerical implementation of periodic boundary condition has been provided in Supplementary Appendix S2.

#### 2.3.2 Volume averaging

In order to represent the RVE as a single value in the macroscopic analysis, it is common practice to compute the volume average of relevant quantities over the RVE. This averaging process allows us to obtain a representative value that captures the overall behavior of the RVE in the macroscopic analysis. By computing the volume average, we can effectively condense the information from the microscale to the macroscale and simplify the analysis at the macroscopic level.

$$\bar{f}=\left\langle f\right\rangle =\frac{1}{V}\int_{V}fdV$$ (21)

here in Equation 21, the volume of the RVE is denoted as V , and the angle brackets ⟨⟩ represent the volume average operation. The quantity f can take the form of a scalar, vector, or tensor and generally exhibits variations at different points within the RVE. The corresponding effective quantity $\bar{f}$ is determined by calculating the volume average of f over the entire RVE volume, resulting in a representative value that characterizes the overall behavior of f at the macroscopic level.

The micro-to-macro scale transition relation is commonly established using the Hill-Mandel condition or macrohomogeneity condition (Hill, 1963; Hill, 1965a; Hill, 1965b; Hill, 1972; Mandel, 1971; Suquet, 1987). This condition ensures that the volume average of the increment or variation of work performed on the Representative Volume Element (RVE) is equivalent to the increment or variation of local work on the macroscopic scale. Expressed in terms of a work conjugated set, which includes the deformation gradient tensor and the first Piola-Kirchhoff stress tensor, the principle of multiscale virtual power, that generalized Hill-Mandel law in a variational setting, can be stated as follows (De Souza Neto et al., 2015):

$$\frac{1}{V^{\eta }}\int_{\Omega_{0}^{\eta }}P^{\eta }:\delta F^{\eta }dV^{\eta }=P:\delta F$$ (22)

The left side of Equation 22 expresses the micro scale and the right side denotes the macro scale, where $\Omega_{0}^{\eta }$ is the microscopic Representative Volume Element (RVE) domain.

Therefore, according to the Hill-Mandel energy consistency relation, considering the specified boundary conditions and since δF is also arbitrary the macroscale first Piola-Kirchhoff stress tensor can be determined as the volume average of the microscale first Piola-Kirchhoff stress tensor.

$$P=\frac{1}{V^{\eta }}\int_{\Omega_{0}^{\eta }}P^{\eta }dV$$ (23)

Considering fibers and matrix domain, Equation 23 can be written as Equation 24

$$P=\frac{1}{V^{\eta }}\left(\int_{\Omega_{0}^{\eta }}P_{m}^{\eta }dV^{\eta }+\overset{N_{f_{\mathrm{Total}}}}{\underset{i=1}{\sum }}\int_{V_{0}^{f_{i}}}P_{c_{i}}^{\eta }dV^{\eta }\right)$$ (24)

where $P_{m}^{\eta }$ and $P_{c_{i}}^{\eta }$ are the first Piola-Kirchhoff stress tensors of the matrix substance and the correction of the $i^{th}$ fiber $f_{i}$ , respectively. To characterize the overall mechanical behaviors of the RVE, volume averaging of stresses and strains was carried out. Equation 25 was employed to estimate the volume-averaged values of the desired outputs.

$${\bar{\sigma }}_{ij}=\frac{1}{V^{\eta }}\int_{V^{\eta }}\sigma_{ij}dV$$ (25)

Here, $V^{\eta }$ is the volume of the element, ${\bar{\sigma }}_{ij}$ and $\sigma_{ij}$ show the average volume values of stress, and the average nodal stresses, respectively. It should be noted that $\sigma_{ij}$ represents the Cauchy stress tensor, which is the true stress acting on the deformed configuration. While the first Piola-Kirchhoff stress tensor P refers to the undeformed (reference) configuration, the Cauchy stress tensor $\sigma_{ij}$ corresponds to the deformed (current) configuration. Both tensors are related through the deformation gradient F , and this distinction is crucial when interpreting the results from the microscale and macroscale analyses.

## 3 Methods

### 3.1 Tissue preparation

**FIGURE 2:**
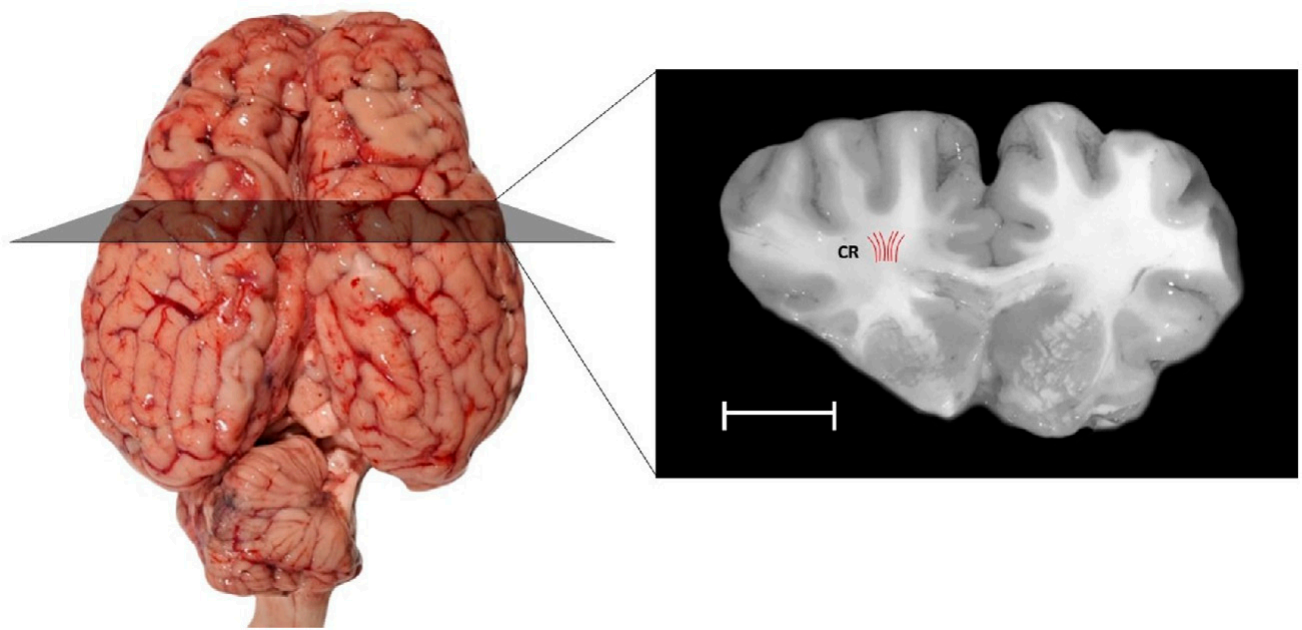
The present study includes samples taken from a specific region of the white matter of the bovine brain: the corona radiata (CR), highlighted in a coronal section. The Red lines indicate the orientations of axonal fibers. Samples were extracted aligned to the direction of axon fibers. The scale bar provides a sense of size: the white scale bar represents 10 mm.

Due to the limited access to fresh human brain tissue and the anatomical similarity between human and bovine brains (Budday et al., 2020; Darvish and Crandall, 2001; Samadi-Dooki et al., 2017), eight fresh bovine brains aged 7 years old were collected from a local slaughterhouse. The brains were kept in 0.01 M Phosphate Buffered Saline solution (PBS) during transportation to the laboratory, and no apparent physical damage was observed (Figure 2). The utilization of this solution ensured that there were no observable alterations in sample shape caused by gravity. To avoid any mechanical decay, they were kept cold at 2°C–4°C after extraction. They were stored at this temperature until shortly before testing. All experiments were performed within 5 hours post-mortem and at room temperature (∼20°C) . Prior to testing, we ensured that each sample was equilibrated to room temperature by allowing sufficient time for temperature normalization. This step was carefully monitored to avoid any rapid temperature fluctuations that could affect the mechanical properties. The transition from cold storage to room temperature was gradual, ensuring sample integrity.

**FIGURE 3:**
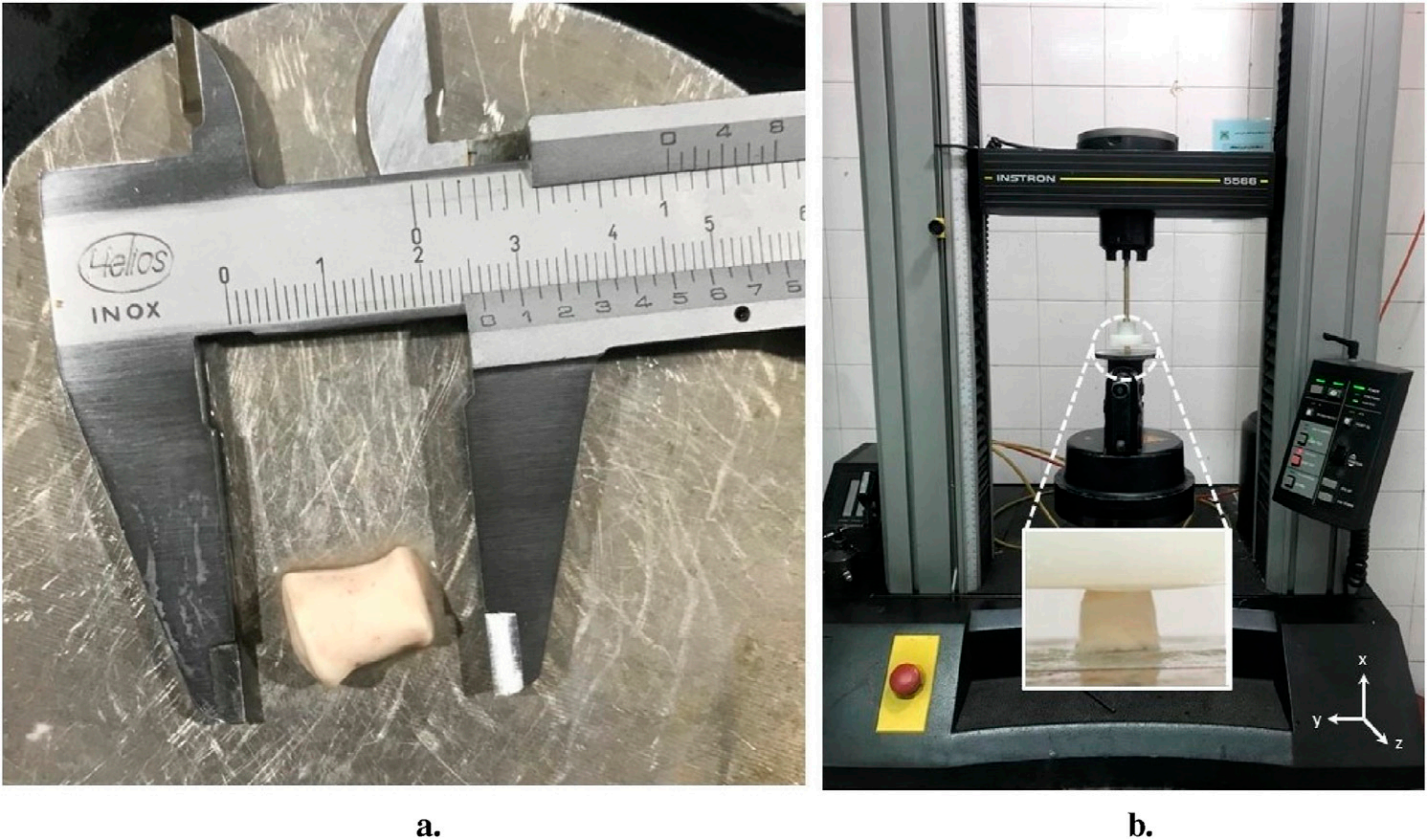
Experimental mechanical test setup; **(A)** An example of cylindrical bovine brain tissue sample obtained from corona radiata(CR) region from the white matter, **(B)** The uniaxial testing device used for the experiment and positioning of the sample within the testing machine.

At first, the cerebellum was removed carefully. Each bovine brain was cut using a surgical scalpel blade. Cylindrical samples (n = 52, where n represents the total number of samples) with a diameter of 10.09 ± 1.68 mm and a height of about 10.5 ± 2.98 mm were extracted by using a cylindrical steel cutter device from the corona radiata (CR) region of white matter as shown in Figure 3A. In order to prevent dehydration, PBS solution was applied to the samples frequently during cutting and before testing. After preparation, each sample was glued to the holders with a thin layer of cyanoacrylate adhesive.

### 3.2 Experimental setup

In order to study the histological changes, 26 out of the 52 samples were randomly selected and divided into two groups: the stress-free control group (n = 13) and the deformed group that was preconditioned (n = 13). The selection was random to ensure an unbiased and comparable distribution of samples across both groups.

The preconditioning contains 3 loading-unloading (tension) cycles up to 10% strain with 1/30 Hz frequency. All mechanical tests were conducted with universal testing machine (Instron 5566 materials testing machine, Instron Co.) as shown in Figure 3B. Uniaxial cyclic loads were applied in the direction of axon fibers in CR (Figure 2). The direction of axon fibers in the corona radiata has been validated with the previous studies (Hoppstädter et al., 2022). We considered quasi-static loading with the speed of v=2 mm/min for the experiments. Considering the fixing protocol, Samples were kept in 4% buffered formalin solution for 72 h.

Furthermore, a dedicated setup was conducted in the experimental study to examine the distinct influence of preconditioning on the mechanical behavior of the tissue. The samples underwent uniaxial tension loading both before and after preconditioning. The initial group of samples underwent uniaxial tension (n = 13), while the subsequent group was preconditioned before undergoing uniaxial tension (n = 13), aimed to assess the mechanical response of the tissue under preconditioning. Uniaxial tension applied was up to 20% strain under 0.033%/s deformation rate. During the tests, PBS solution was continuously sprayed onto the specimens.

### 3.3 Histological investigations

**FIGURE 4:**
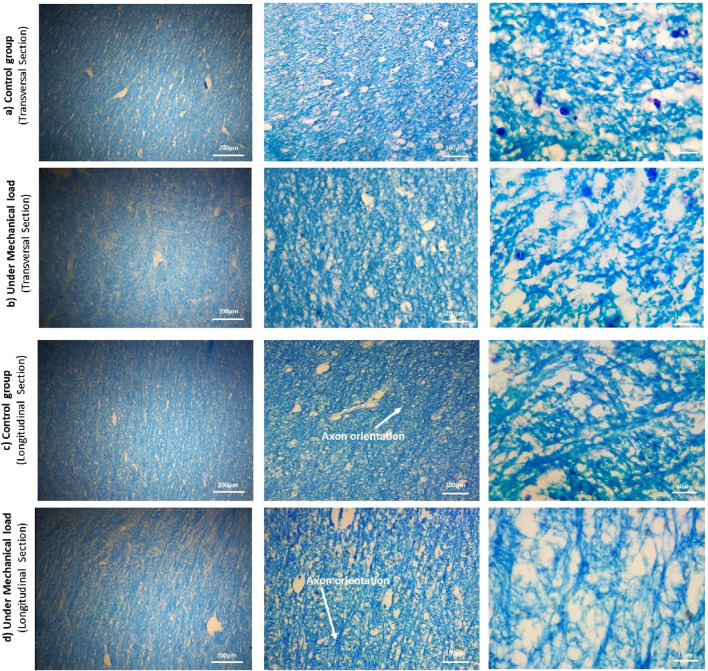
The microstructure of the samples of the control and preconditioned groups from the CR region of the cow brain, cut in two directions: perpendicularly and along the length of the axon fibers, using the Luxol fast blue staining method. The magnification increases from right to left: 20x, 100x, and 200x. The cross-sectional images show the diameter and position of the axons, and the longitudinal section images show the orientation of the axons.

Histological studies were performed on twenty-six samples, carefully selected to represent both control and preconditioned groups (n = 13 for each group), to determine the relationship between microstructural changes and mechanical behavior. In order to analyze the histology, the samples were cut in two directions, perpendicular to the axons and the longitudinal direction of the axons. The dual cutting directions were employed to ensure a comprehensive analysis of axonal alignment and structural integrity, as different orientations can reveal distinct aspects of tissue architecture. Sections were stained with Luxol Fast Blue protocol to visualize axonal fibers. After that, all histological sections were digitized using a microscope equipped with 20, 100, and 200 X objectives: first, snapshots of the samples were scanned. Samples were randomly selected for cutting to avoid artifacts that could distort the analysis, such as holes (Figure 4).

Due to the limitations and difficulty of the mentioned tissue staining method, dark blue spots were seen in the images, which are color deposits. In the transverse sections, after identifying the axons using ImageJ v1.52 software (Rueden et al., 2017), the histological images at scale 20 were analyzed by QuPath v0.4.3 software (Bankhead et al., 2017) to calculate the diameter of the axons. The results of these calculations are shown in Table 1. Additionally, in the longitudinal sections, the orientation angles of axons were randomly measured to assess alignment and potential transverse axons. Figure 4 shows the results of the distribution of axonal fibers both in transverse and longitudinal sections.

**TABLE 1:** Basic Statistics of axon diameters of the bovine corona radiata (CR) for two groups: control and preconditioned, obtained from histological image analysis.

Group	N ± SD	Mean ± SD( μ m)	Max-Min( μ m)	Median( μ m)	Variance
Control	115 ± 3	0.8646 ± 0.238	1.2095	0.8223	0.0634
Preconditioned	98 ± 2	0.8637 ± 0.251	0.9525	0.8615	0.0567

Regarding the presence of transverse axons in the longitudinal sections, we observed that the number of axons running transversely was minimal, accounting for approximately 2% of the total axons in the longitudinal sections. Given their small proportion, we considered their contribution to be insignificant in influencing the overall mechanical response. This observation was consistent across all samples and suggests a strong alignment of axons along the primary direction, which could be relevant under certain loading conditions. The axonal volume fractions for the two control and preconditioned groups were 32.106 ± 1.05 and 27.765 ± 0.97, respectively, obtained from transverse-sectional histological images.

## 4 Micromechanical modeling framework

In this study, the statistically representative volume element (RVE) used was a cubic grid containing a random distribution of axons, with their primary orientation in the X direction, in the extracellular matrix (ECM). The density of axons is defined using the volume fraction ( $V_{f}$ ) determined from histological images, defined in the model as the ratio of fiber volume to RVE volume, as shown below (Yousefsani et al., 2018b):

$$V_{f}=\frac{\pi }{4a^{2}}\overset{N}{\underset{i=1}{\sum }}d_{i}^{2}$$ (26)

In Equation 26, a represents the edge length of the lattice, $N_{i}$ is the number of fibers, and $d_{i}$ is the diameter of the ith cylindrical fiber. A fiber architecture with random position, diameter, and orientation was created in the randomization procedure.

The randomization of axonal diameters was achieved using a cumulative density function (CDF) based on a generalized extreme value distribution function (GEV). The GEV distribution was chosen due to its demonstrated superiority in fitting empirical data. A recent study compared various parametric probability distributions against axon diameter data from electron microscopy and found that the GEV distribution consistently outperformed other models, including the commonly used gamma distribution. This was attributed to its ability to better capture key characteristics of axon diameter distributions, such as the location and scale of the mode, as well as the behavior of distribution tails. Other distributions, such as the inverse Gaussian, lognormal, loglogistic, and Birnbaum-Saunders, also provided better fits than the gamma distribution but were not as accurate as GEV in describing distinct subpopulations of axons (Sepehrband et al., 2016).

**FIGURE 5:**
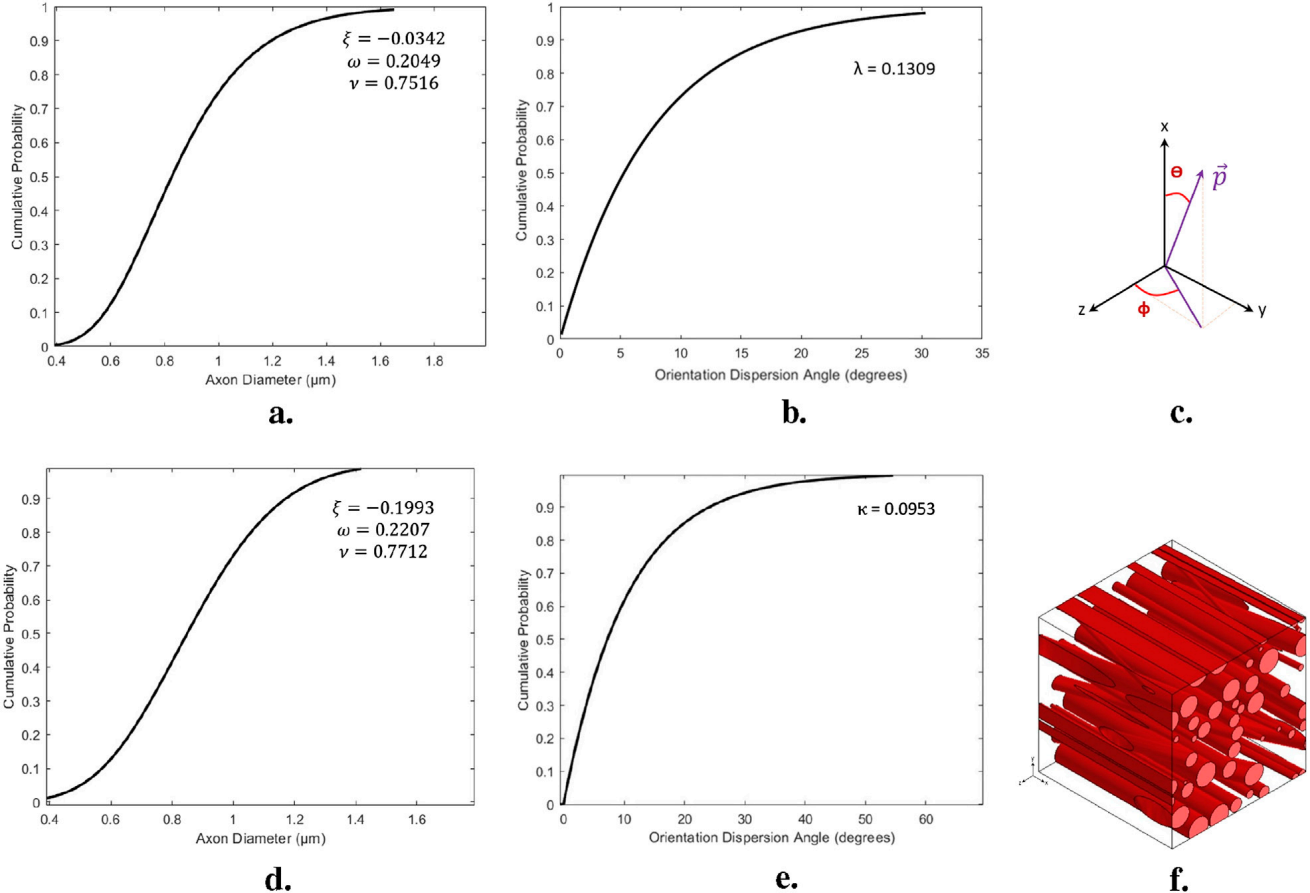
Process of construction the statistical volume element; **(A, B)** The cumulative distribution functions of axonal diameters and orientations presented for a representative structure of white matter, specifically the CR for the Control group. **(C)** The spherical components of the orientation vector $\vec{p}$ assigned to the axons within the RVE model. **(D, E)** The cumulative distribution functions of axonal diameters and orientations presented for a representative CR structure for the Preconditioned group. These distributions are based on experimental histological data analysis from the present study. **(F)** Schematic of a histology-informed RVE featuring random positions, diameters, and orientations of axons.

This CDF was parameterized with a shape factor ξ , the location parameter ν that could be any real number, and a scale factor ω to describe the density of axonal diameters. Note that these parameters should not be confused with material parameters. The probability density function (PDF) of the mentioned distribution defined as shown in Equations 27, 28 (Figures 5A, D):

$$g\left(y\right)=\frac{1}{\omega }t{\left(y\right)}^{\xi +1}e^{-t\left(y\right)}$$ (27)

where

$$t\left(y\right)=\left\{\begin{array}{cc} {\left[1+\xi \left(\frac{y-\nu }{\omega }\right)\right]}^{-\frac{1}{\xi }},\; & \xi \neq 0\\ \;\\ e^{-\left(\frac{y-\nu }{\omega }\right)},\; & \xi =0 \end{array}\right.$$ (28)

to randomize the orientations of the axons, another CDF was obtained from histological images. The CDF was modeled using an exponential fit with a rate parameter represents in Figures 5B, E.

This cumulative density function describes the distribution of axonal orientations, which are then used to determine the spherical components of the orientation vector $\vec{p}$ assigned to the axons within the RVE model. Therefore, Equation 29 indirectly influences the orientation vector $\vec{p}$ by modeling the probability distribution of orientation angles, ensuring a realistic randomization of orientations for the axons.

$$H\left(y;\kappa \right)=1-e^{-\kappa y}$$ (29)

The orientation vector $\vec{p}$ was assigned to the axons in the statistical RVE, characterized by two spherical angles θ and ϕ . The angle θ represented the angle between the primary axonal orientation (aligned with the X-axis) and vector $\vec{p}$ which was determined based on histological analysis. The angle ϕ represented the angle between the Z-axis and the projection of the orientation vector $\vec{p}$ onto the YZ-plane, assumed to follow a generally distributed range from zero to 360° (Figure 5C).

Once an appropriate sampling method was employed and the basic parameters characterizing the microstructural features were extracted from the histological images, the two groups of the RVEs: before and after preconditioning, were created. To avoid fiber overlap, a minimum distance of 0.05 μm is set as an acceptable threshold between the outer diameters of neighboring fibers. While the Digimat software (Digimat-FE Toolkit) offers the ability to place fibers one by one in random positions on the unit cell plane, it was used to generate the RVEs. Positioning of axonal fibers in the RVEs continued until the desired volume fraction was achieved. The obtained effective volume fractions are shown in Tables 2, 3 for each RVE size in two groups. Due to the geometric complexity of the statistical volume element, as mentioned, with the random distribution, diameter, position, and orientation of the axon fibers, Digimat-FE was exclusively used to generate the geometry. Then, the generated geometry was exported as a set of Parasolid files to Abaqus software.

**TABLE 2:** Number of axons and effective volume fraction For the edge size sensitivity analysis of the RVE related to the Control group.

RVE size ( μ m)	4	5	6	7	8	9	10	11	12	13	14
NO. of inclusions	4	6	11	15	23	34	41	53	67	76	83
Effective volume fraction	0.318	0.338	0.320	0.322	0.315	0.323	0.338	0.327	0.323	0.332	0.323

**TABLE 3:** Number of axons and effective volume fraction For the edge size sensitivity analysis of the RVE related to the Preconditioned group.

RVE size ( μ m)	4	5	6	7	8	9	10	11	12	13	14
NO. of inclusions	3	5	9	12	19	27	34	44	56	62	69
Effective volume fraction	0.292	0.275	0.279	0.284	0.277	0.271	0.282	0.276	0.270	0.282	0.271

Geometric periodicity and periodic boundary conditions (PBC) were applied to all faces of the volume element, ensuring periodicity in the displacement field flux. This involves relating the degrees of freedom of nodes on one face to those on the opposite face. While PBC offers more accurate predictions than Dirichlet and mixed boundary conditions, it also increases computational demands due to the many constraint equations required. The resulting RVE, with random axon positions, diameters, and orientations, is shown in Figure 5F. The mechanical behavior of axons and the ECM is described using an Ogden-type strain energy density function, and the embedded element technique (EET) has been used to enhance mesh quality (Pan et al., 2013).

**FIGURE 6:**
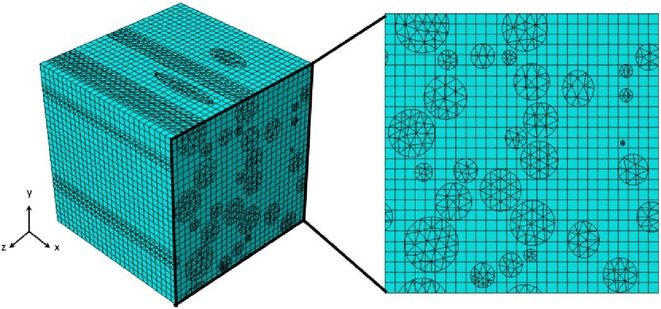
Representation of RVE applying Embedded Element Technique with independent mesh grids for the host and guest domains.

For Ogden hyperelastic materials, the surplus strain energy can be eliminated by appropriately adjusting the stiffness of the axons, as represented in Equation 14. To model the axonal fibers, 4-node linear tetrahedral composite elements (C3D10H) were used, while 8-node linear brick hybrid elements (C3D8RH) were used to represent the ECM, considering incompressibility. Face partitioning into host and guest domains was applied to maintain mesh symmetry in opposite nodes of the models shown in Figure 6. A custom Python script applied constraints to the models in ABAQUS. The embedded element technique was used to facilitate PBC since the grids in both host and guest domains were independent and thus had identical sizes, shapes, and elemental distributions. To ensure stress continuity (elastic boundary conditions), simulations confirmed the symmetry of stress distribution on opposite faces of the RVE models (Hill, 1963).

### 4.1 Optimization procedure

A multi-objective optimization procedure determined the hyperelastic constants for the axonal fibers and ECM from experimental mechanical data in an inverse manner. Assuming quasi-static large deformation in the axonal direction, the cost functions were defined as the deviations between the homogenized responses of the RVE and the experimental data. The search for optimal hyperelastic material constants, which minimized the cost functions, was performed using an evolutionary optimization procedure known as the imperialist competitive algorithm (Atashpaz-Gargari and Lucas, 2007). Table 4 depicts a flowchart outlining the optimization process.

**TABLE 4:** The optimization procedure in the present study.

Start	
1	Initial sets of material parameters $\left(\mu_{\mathrm{Axon}},\mu_{\mathrm{ECM}},\alpha \right)$
2	Run FE Simulation and extract related model stresses
3	Get stress data from experiment
4	Formulate Cost Function used values from experiment and FE model to minimize the differences between RVE and experimental stresses
5	If: 0≤Cost≤0.2 , use ICA to generate new set of material parameters $\left(\mu_{\mathrm{Axon}},\mu_{\mathrm{ECM}},\alpha \right)$ , go to 2
6	Else: Exit from the Optimization procedure
End	Optimal set of hyperelastic material parameters

The optimization process identifies three independent variables: $\mu_{\mathrm{axon}}$ , α and $\mu_{\mathrm{ECM}}$ . The algorithm employed an initial population size of n=100 sets of variables, with an assimilation coefficient of γ=0.3 . Among these sets, the ten most effective solutions were chosen as imperialists during the first iteration, forming the basis of 10 initial empires. To reduce simulation costs, all variables were discretized within specifically limited boundaries, considering the values reported in the literature (Budday et al., 2017; Meaney, 2003; Yousefsani et al., 2020; Hoursan et al., 2020; Chavoshnejad et al., 2021; Yousefsani et al., 2018a). The boundaries for the variables are defined as follows: $\mu_{\mathrm{axon}}\in \left[100,2000\right]$ Pa, $\mu_{\mathrm{ECM}}\in \left[1,500\right]$ Pa and α∈[−30,30] except [−1, 0, 1]. The cost function was formulated as the sum of the coefficient of determination (R^2^) and the Root Mean Square Error (RMSE) of stress values between every two sets of data points to account for deviations of the patterns and the values of the resulting curves from the experimental data, represents in Equation 30.

$$\begin{array}{cc} Cost= & \sqrt{\frac{1}{N}\overset{N}{\underset{i=1}{\sum }}{\left(\frac{\sigma_{i}^{M}-\sigma_{i}^{E}}{\sigma_{i}^{E}}\right)}^{2}}\\ & +\frac{\overset{N}{\underset{i=1}{\sum }}{\left(\sigma_{i}^{M}-\sigma_{i}^{E}\right)}^{2}}{\overset{N}{\underset{i=1}{\sum }}{\left(\sigma_{i}^{E}-{\bar{\sigma }}_{i}^{E}\right)}^{2}} \end{array}$$ (30)

While N indicates the quantity of data points in the stress curve. The nominal stress is denoted by σ , and i is the index used for summation. The values obtained from the models and experiments are labeled as M and E, respectively. Equation 31 provides a description of the mean stress present in the experimental curve.

$${\bar{\sigma }}^{E}=\frac{\overset{N}{\underset{i=1}{\sum }}\sigma_{i}^{E}}{N}$$ (31)

The stop condition, which determines the optimal set of parameters, was defined as having only one remaining empire with a total cost of 0≤Cost≤0.2 . If this condition was met, the resulting optimal parameters were checked to ensure they fell within the initial range assumed beforehand, and the process was ended. If not, a new set of parameters was created, and the competitive algorithm was repeated until the stop condition was satisfied. To ensure that the best global solutions were obtained, a sensitivity analysis was performed to confirm that the optimal solutions were not affected by the upper and lower bounds of the parameters.

### 4.2 Sensitivity analysis

As explained, EET utilizes a host mesh consisting of 3D elements, with guest elements embedded through a kinematic bond created by the shape functions of the continuum mesh. To examine how the mesh size affected the responses of the RVE model, a quasi-static extension λ=0.2 was applied in the axonal direction (X-axis in Figure 6). The number of nodes in the models varied from approximately 1,000 to 39,000 (Figures 8A, B). The RVE models had an effective axonal volume fraction $\left(V_{f}\right)$ of 32% and 27% for both before-preconditioning and after-preconditioning groups, respectively. Tables 2, 3 provide information on the number of axons and the effective volume fraction of the guest domain for the RVE models of two groups. The investigation included examining the homogenized overall and localized responses regarding the normalized maximum stress $\left(\sigma_{\max}/\sigma_{\mathrm{avg}}\right)$ , as depicted in Figure 8.

**FIGURE 7:**
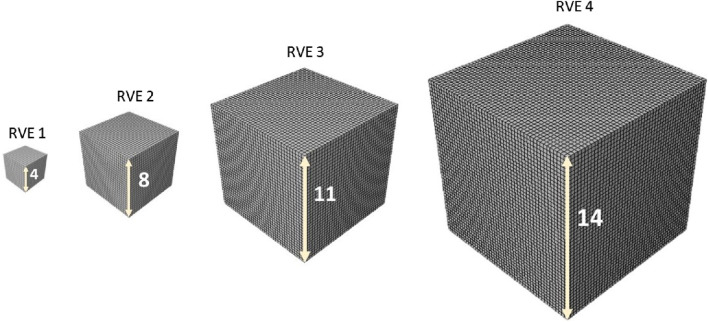
3D host meshes employed to model the matrix substance. The same element size was used for all RVEs.

After using the same optimal grid size for RVEs, in a parametric study, the influence of the minimum edge size on the response of the statistic RVE models was investigated (Figure 7). The models were subjected to the same loading conditions as in the mesh sensitivity analysis. While the mesh size of both models remained fixed at the optimal value, the lattice edge size was varied from 4 to 14 μm . To provide a general overview of the procedure, four RVEs are chosen and illustrated in Figure 7. These RVEs, selected from a range of 4 μm to 14 μm , are chosen according to a specific ratio that reflects their relative enlargement within this range.

**FIGURE 8:**
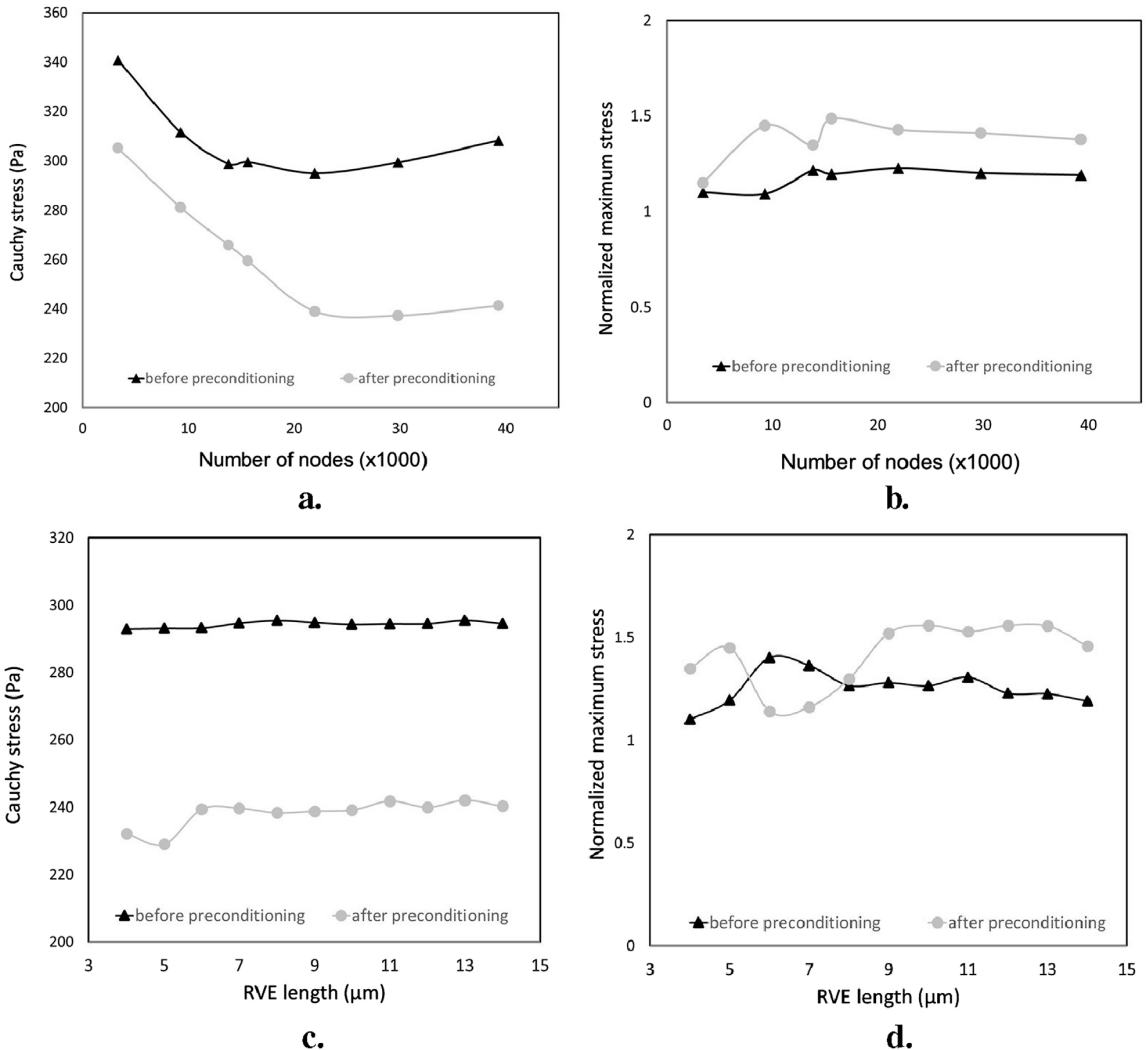
Sensitivity analysis of the overall and local responses of the RVE models for two groups: before preconditioning and after preconditioning. **(A, B)** the effect of the grid size; **(C, D)** the effect of the RVE edge length.

The effect of the edge size on the overall and local response is illustrated in Figures 8C, D. As such, an edge length of 8 μm and 10 μm were chosen for before and after RVE model sizes, respectively.The orientation vector $\vec{p}$ was assigned to the axons in the statistical RVE, characterized by two spherical angles θ and ϕ . The angle θ represented the angle between the primary axonal orientation (aligned with the X-axis) and vector $\vec{p}$ which was determined based on histological analysis. The angle ϕ represented the angle between the Z-axis and the projection of the orientation vector $\vec{p}$ onto the YZ-plane, assumed to follow a generally distributed range from zero to 360° (Figure 5C).

## 5 Results

**FIGURE 9:**
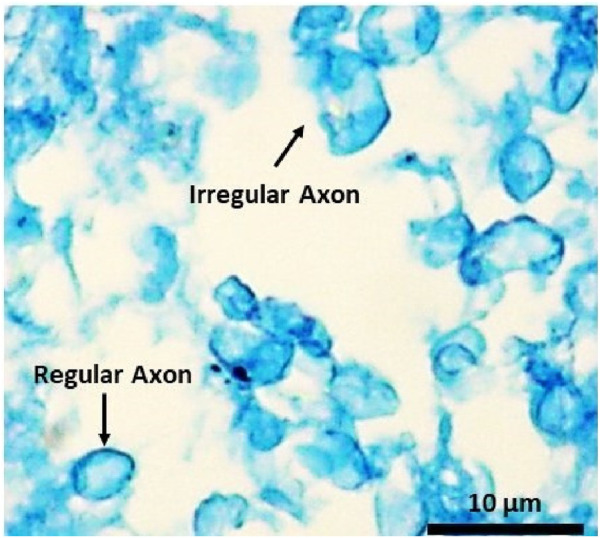
Histological image of the transverse section of the white matter’s CR region shows regular and irregular axons of the preconditioned sample.

**FIGURE 10:**
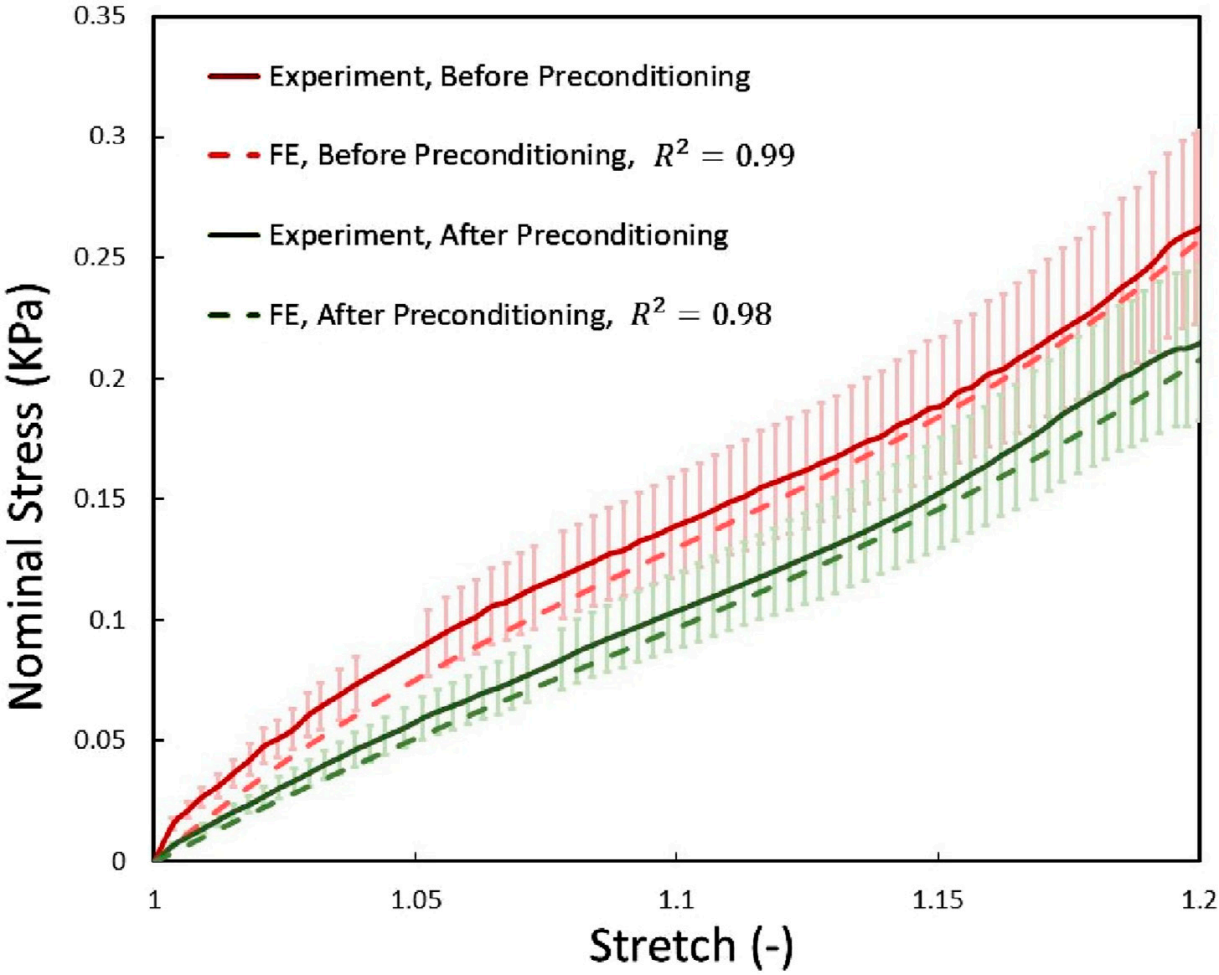
The mechanical response of the two before and after preconditioning groups of RVEs using optimized material properties were compared with the experimental data obtained from the current study under quasi-static tensile load. Error bars represent standard deviations of experimental tests.

Initially, we investigated the mechanical responses of bovine brain tissue through experiments to study the effect of preconditioning at the macro and micro scales. The mechanical response of bovine brain tissue at the macro scale, before and after preconditioning, is illustrated in Figure 10. The preconditioned group exhibited a lower peak stress compared to the control group, indicating a softening of the tissue following preconditioning. In contrast, the control group showed greater stiffness, suggesting that preconditioning reduces the tissue’s resistance to mechanical loading, a finding consistent with previous research on soft tissues Budday et al. (2020). Figure 9 shows the transverse section of axonal fibers after preconditioning from CR, where normal and damaged axons are shown. Accordingly, irregular axons appeared with geometrical changes. We observed abnormality of axon geometry in Figure 9, but the damage considered in this study is the difference in the number of axons analyzed by histological staining method. Statistical and histological analysis shows that the volume fraction for the control group was approximately 32%. For the preconditioned group, it was about 27%. The experimental study showed that preconditioning leads to the damage of several axon fibers in the specific area of the myelin sheath, so by comparing the histological images of the two groups, the observed axons are reduced by about 17%. This is due to the non-staining of more damaged axons in the preconditioned group.

As shown in Figure 5, we studied the effect of macroscopic loading-unloading cycles on the microstructure of brain tissue using multiscale models based on representative volume elements (RVEs) that include a random distribution of axonal fibers in the extracellular matrix. Statistical RVE was developed based on probability distribution functions obtained from histological observations. The dispersion of axonal fibers in the white matter of brain tissue necessitates the inclusion of multiple fibers in the RVE to represent the microstructure accurately. In the micromechanical finite element model, two independent domains of matrix and axon fibers are modeled as three-dimensional elements using the embedded element technique as represented in Figure 6. Based on histological results, we simulated two groups of RVE similar to the white matter microstructure of brain tissue before and after preconditioning. Each RVE is subjected to a uniaxial strain of up to 20% in the small deformation regime based on the overall mechanical properties of CR using periodic boundary conditions as one of the classical multiscale conditions.

To analyze the effect of mesh size on the responses of the two groups of RVE models, Homogenized cauchy stresses and normalized maximum stresses were studied for both groups of RVE models. The results showed that local and overall tissue responses were approximately independent of the mesh size when the elements were sufficiently fine. However, the localized responses of the RVE model after preconditioning were more sensitive to the grid size, requiring more nodes to converge compared to the RVE model before preconditioning (approximately 19,000 and 24,000 nodes for before and after preconditioning groups, respectively). The RVE model of the after-preconditioning group also exhibited higher fractions of local stress and relatively lower values of homogenized Cauchy stress than the RVE model due to the increasing dispersion of axonal orientations in the guest domain.

By fixing the RVE mesh size at the optimal value, we varied the grid edge size of these models from 4 to 14 μm , as shown in Figure 7; Tables 2, 3. Show the number of axons and each model’s effective volume fraction of the guest domain. The RVE models were subjected to the same loading conditions before and after preconditioning. The homogenized response of both groups was more sensitive to the lattice edge size. Both the homogenized and local responses of the RVE model showed considerable variations with changes in the edge length; however, marginal local variations in the maximum stress were higher in after preconditioning RVE. since changes in the arrangement of axonal fibers result in microscopic stress fields showing significant heterogeneity, The minimum acceptable edge length increased with the microstructural randomness level, requiring a larger RVE model compared to the RVE model from before preconditioning group to stabilize tissue homogenized and local behaviors against microstructural variations. Ultimately, the RVE model before preconditioning was meshed with almost 20,000 nodes, while the RVE model after preconditioning required approximately 27,000 nodes, and their respective lattice edge sizes were 8 μm and 10 μm .

To determine the hyperelastic material properties of axon fibers and ECM, we characterized them by fitting the homogeneous response of RVE on our experimental results based on the ICA algorithm (Table 4). For both models, we set the volume fraction observed in the tissue through histological observations, as stated before. The optimization process converged after 91 iterations for the RVE model for before the preconditioning group and 142 iterations for the RVE model for after the preconditioning group model, with total runtimes of about 5 and 8 h, respectively, on a computer with 6 CPUs and 16 GB of RAM. Table 5 shows the optimal hyperelastic constants suggested by previous studies. Table 6 shows the optimal hyperelastic constants for the present study and the provided corresponding values of the Cost functions.

**TABLE 5:** The obtained optimum material constants reported in previous studies.

	$\mu_{\mathrm{axon}}$ (Pa)	$\alpha_{\mathrm{axon}}$	$\mu_{\mathrm{ECM}}$ (Pa)	$\alpha_{\mathrm{ECM}}$	References
Chavoshnejad et al	722	−23	110	−6	
	110	−6			Chavoshnejad et al. (2021)
Hoursan et al. (RVE)	1,062.78	4.89	80.12	4.89	Hoursan et al. (2020)
Hoursan et al. (SVE)	738.3	4.49	99.36	4.49	Hoursan et al. (2020)
Yousefsani et al	1,130.3	4.91	87.4	4.91	Yousefsani et al. (2018a)
Pan et al	33,280	8.22	11,093	8.22	Pan et al. (2013)
Meany	290.82	6.16	-	-	Meaney (2003)
Saeidi et al	80.8	62.3	353.5	−21.5	Saeidi et al. (2023)

**TABLE 6:** The optimal hyperelastic material constants of white matter constituents obtained for the models before and after preconditioning, in the present study.

	$\mu_{\mathrm{axon}}$ (Pa)	$\alpha_{\mathrm{axon}}$	$\mu_{\mathrm{ECM}}$ (Pa)	$\alpha_{\mathrm{ECM}}$	Minimum cost $\left[R^{2}\;RSME\right]$
Control	796.4	20.65	85.61	20.65	0.1672 [0.975 0.121]
Preconditioned	598.4	21.55	98.97	21.55	0.1858 [0.961 0.197]

To calibrate the hyperelastic RVE models and their associated material constants, we compared the predicted mechanical behavior of the CR, a white matter structure, with experimental data. We compared the homogeneous responses of the models under quasi-static longitudinal expansion with the experimental results, as shown in Figure 10. Results were presented in terms of nominal stress to align with experimental data. We observed good agreement between the predicted and experimental stress curves for corona radiation, with normalized RMSEs below 5% and 7% for the RVE models, respectively.

## 6 Discussion

While numerous studies have investigated the nonlinear mechanical behavior of brain tissue under various loading conditions, providing a broad understanding of its macroscopic properties (Jin et al., 2013; Budday et al., 2017; Franceschini et al., 2006; Shuck and Advani, 1972; Arbogast and Margulies, 1998; Bilston et al., 2001; Darvish and Crandall, 2001; Hrapko et al., 2008; Rashid et al., 2013; Destrade et al., 2015), gaps remain in understanding the microstructural changes that occur under preconditioning. Existing research has predominantly focused on characterizing the mechanical properties of brain tissue *ex vivo*, with limited exploration of how *in vivo* conditions and microstructural changes influence these properties, particularly after preconditioning (Budday et al., 2020; Miller and Chinzei, 2002; Velardi et al., 2006). Preconditioning is known to alter tissue behavior, such as reducing stiffness, yet its influence on the brain’s internal fiber arrangements remains poorly understood (Fung, 1993; Cheng et al., 2009; Prevost et al., 2011; Gefen and Margulies, 2004). This study contributes to filling this gap by integrating experimental data with a micromechanical finite element model, enabling a detailed examination of how preconditioning affects the tissue’s microstructure. Our findings provide insights into the interplay between microstructural changes and macroscopic mechanical responses, offering a deeper understanding of the mechanics of brain tissue under cyclic loading and advancing current knowledge in this field.

Initially, our experimental protocol involved dividing cylindrical samples of brain white matter tissue (from CR) into two groups: control and preconditioned. The control group remained stress-free, preserved in formalin, while the preconditioned group underwent mechanical testing based on the designed protocol. Tensile loading, up to 20% strain under quasi-static conditions, was applied before and after preconditioning to explore how cyclic loading impacts the mechanical properties of brain tissue. Preconditioning involved three tension cycles applied along the axonal fiber direction.

As shown in Figure 10, preconditioning significantly reduced tissue stiffness, consistent with earlier studies that observed a softer tissue response post-preconditioning (Budday et al., 2020). This reduction suggests that preconditioning alters tissue mechanics in a repeatable manner. One prevailing explanation for this softening, supported by previous research (Franceschini et al., 2006; Budday et al., 2017), is the gradual fluid drainage from brain tissue, which decreases resistance to loading. However, our findings indicate that microstructural changes, particularly in the arrangement and potential damage to axons, may also contribute significantly to the observed mechanical behavior, providing a novel perspective on brain tissue mechanics under preconditioning.

To further investigate these microstructural changes, we preserved specimens post-testing for histological analysis. Representative tissue samples were carefully selected to avoid cutting artifacts (such as holes), and Luxol Fast Blue staining confirmed the integrity of the myelin sheaths in both control and preconditioned groups. Artifacts identified as non-specific staining did not compromise the staining accuracy. Quantitative analysis of axonal properties, such as diameter and orientation, revealed consistent measurements across both groups, with an average axon diameter of approximately 0.86 μm . As noted in Table 1, the GEV (Generalized Extreme Value) distribution provided the best fit for axon diameter data, aligning with prior studies (Sepehrband et al., 2016).

Notably, the explicit difference was the number of damaged axons in the preconditioned group. This observation somehow contrasts with prior studies that reported no permanent tissue changes from preconditioning (Budday et al., 2017; Budday et al., 2020). The lack of sufficient histological data in those earlier studies may explain the discrepancy, as our results suggest that axonal damage, detectable through Luxol Fast Blue staining, plays a critical role in the tissue’s altered mechanical response. Under microscopy, damaged or demyelinated axons, which appeared pale or unstained, provided clear evidence of microstructural disruption, highlighting a significant consequence of preconditioning that warrants further investigation (Carriel et al., 2017; Sargon et al., 2007).

Mechanical testing of brain tissue poses significant challenges, particularly due to the specimen’s ultra-soft nature. Attaching samples to holding surfaces introduces potential boundary effects that may result in non-uniform deformation, compromising the assumption of homogeneity. Gravitational forces acting on cylindrical samples further contribute to inhomogeneities, potentially affecting the preconditioning process. Moreover, the inherent limitations of measurement accuracy under various loading conditions highlight uncertainties regarding whether these deformations mimic physiological conditions *in vivo*. The *ex vivo* mechanical environment and the deformations induced by tensile loading differ from natural *in vivo* conditions, where brain tissue is well-preserved and less susceptible to such distortions. During sample preparation and handling, brain tissue often deforms, leading to changes in shape that can affect the results. These deformations, such as loss of cylindrical integrity, introduce minor discrepancies in sample dimensions, which can influence tissue behavior under loading. Despite efforts to average results across samples, variations in sample height and non-uniform specimen dimensions introduce challenges in interpreting the true extent of microstructural changes. For instance, subtle asymmetries in specimen mounting may result in misleading anisotropic responses, obscuring the tissue’s actual mechanical properties.

Post-mortem changes in brain tissue properties have been documented, with studies on porcine brain tissue indicating a noticeable increase in stiffness as early as 6 h after death (Nicolle et al., 2004; Garo et al., 2007). In contrast, tests on bovine brain tissue suggest that stiffness remains relatively unchanged between 2 h and 5 days post-mortem (Darvish and Crandall, 2001; Budday et al., 2015). All mechanical tests in this study were conducted at room temperature, acknowledging that temperature likely affects tissue biobonds. However, the primary goal of this research was to investigate the relationship between tissue microstructure and mechanical behavior, which limited our focus on the impact of temperature. While temperature effects remain an important factor, addressing them was beyond the scope of this study due to the additional complexity and cost involved.

We recognize the inherent challenges of conducting mechanical testing on brain tissue, and the results may not fully capture the material’s precise mechanical properties. This study, however, focused on identifying the microstructural changes in axon arrangement before and after preconditioning, providing valuable insights into how these changes impact tissue mechanics. While uncertainties in mechanical testing may limit the accuracy of some behavioral parameters, our approach reveals new and significant information about the microstructural alterations induced by preconditioning. For this investigation, white matter samples were extracted exclusively from the corona radiata region to ensure consistent alignment of axon fibers. The potential impact of regional differences within brain tissue structures was not considered, representing a limitation of this study. Future research should investigate microstructural variations across different brain regions to offer a more comprehensive understanding. Additionally, this study focused on the hyperelastic behavior of brain tissue during slow deformations, such as brain development or tumor growth (Budday et al., 2014). While brain tissue is known to exhibit time-dependent behaviors, such as viscoelasticity and stress relaxation due to interstitial fluid interactions, these aspects were not addressed in our current model, presenting another avenue for future work.

The highly nonlinear mechanical behavior of brain tissue stems from its complex and heterogeneous microstructure. Understanding the mechanisms of axonal injury in brain white matter requires the development of advanced models that account for this intricacy. This study introduces a micromechanical model, represented by a statistical representative volume element (RVE), which captures the arrangement of axonal fibers through histological analysis before and after preconditioning. By incorporating microstructural characteristics, including axon orientation, volume fraction, and diameter, our model enables a more accurate depiction of localized stresses within the tissue substructure. This approach offers deeper insights into the mechanical behavior of white matter, particularly under varying loading conditions, and enhances the understanding of how preconditioning influences its mechanical properties. The orientation of axon fibers in white matter is neither perfectly aligned nor entirely random, but follows a distribution within specific ranges (Wang et al., 2023; Lee et al., 2014).

In this study, the effect of changes in the orientation of axonal fibers on the mechanical behavior of brain tissue was investigated (Figure 5). For this purpose, other microstructural parameters of axonal fibers, such as volume fraction and diameter of axons, were determined from transverse-sectional images. Specifically, considering the effective volume fraction in each statistical characteristic volume for each group, the diameters of the fibers were randomly distributed from 0.42 to 1.638 μm for the control group, from 0.45 to 1.4 μm for the preconditioned group in the volume element. Then, according to the orientation distribution of axon fibers, the alignment of fibers was applied to the geometry of the model. The loading direction was the X-axis, while the range of fiber orientation was measured from 0 to 31° for the control group and from 0 to 54.5° for the loaded group, and their distribution was random. The deformation fields of RVEs are calculated by Abaqus finite element software v.2021. Also, we calculate the general mechanical responses of RVEs in tensile loading in order to calibrate the model with experimental data.

**FIGURE 11:**
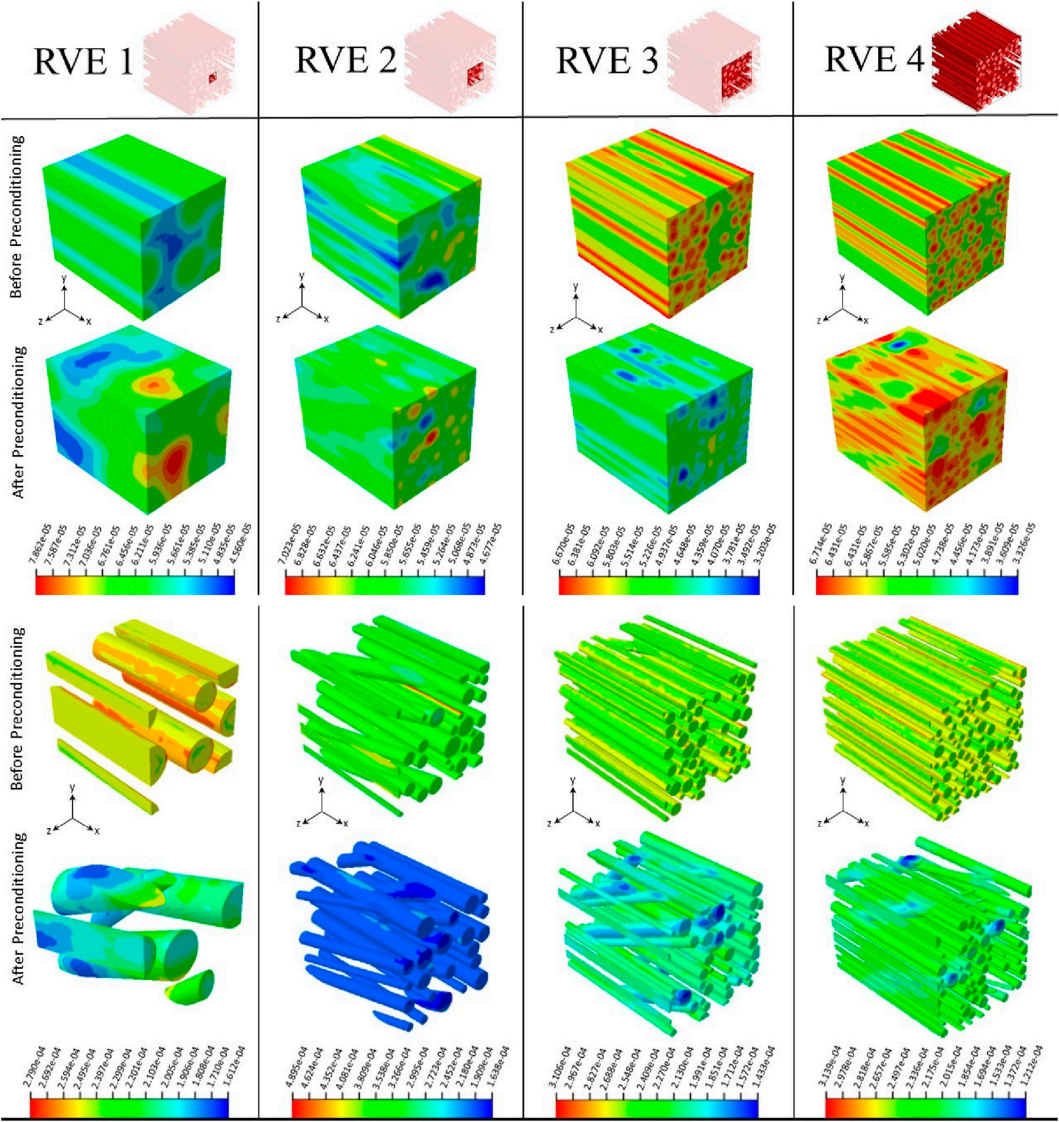
Deformed configurations and normal stress (S11) contour plots show RVE models in two before and after preconditioning groups under 20% quasi-static longitudinal tension, respectively. Configurations are shown separately for host and guest domains. Stress values are expressed in mega pascals (MPa). RVE size increases from left to right.

Figure 11 shows the mechanical behavior of the statistical characteristic volume of the brain tissue for the before and after preconditioning groups subjected to tensile load and periodic boundary conditions. It shows the reduction of stress in the fibers with the increase of the orientation angle of the axonal fibers under preconditioning. The corresponding RVE shows a softer overall mechanical response, which is in line with the experimental data (Figure 10). Also, on the same basis, Figure 11 shows that axonal fibers with a smaller angle between the direction of the fiber and the direction of loading in the control group are subjected to greater tension, which leads to a stiffer overall mechanical response. The trend of microscopic stress changes is consistent with changes in the overall mechanical response. The mechanical behavior of the brain tissue depends on the orientational distribution of the axonal fibers, in addition to the volume fraction with respect to the loading direction. Therefore, the significant difference between the reported mechanical behaviors could be caused by the orientation distribution of the fibers. Recent experiments with Ogden’s hyperelastic formulation showed that, in general, the shear modulus (μ) is direction-dependent, but the characteristic nonlinear behavior of the material for axons and the extracellular matrix, i.e., the parameter α , is not sensitive to the direction of the test (Meaney 2003; Yousefsani et al., 2018a; Yousefsani et al., 2018b). As a result, the stress-strain relationship is set based on $\alpha =\alpha_{\mathrm{Axon}}=\alpha_{\mathrm{ECM}}$ , so three independent parameters $\mu_{\mathrm{ECM}}$ , $\mu_{\mathrm{Axon}}$ and α are sufficient to describe the hyperelastic properties of white matter components (Hoursan et al,. 2020; Yousefsani et al., 2018a; Yousefsani et al., 2018b). The α parameter is the strain magnitude-sensitive nonlinear characteristic of the tissue, and we assume equality for axons and ECM.

The hyperelastic material constants obtained in our study for axons and ECM, based on the histology-based RVE model, generally resemble those suggested by previous studies that considered more histological details (Table 5). The shear modulus of the axons for the preconditioned group decreased by 24% from the control group. Considering previous studies, the axon shear modulus for the control group was 2.7 times, and for the preconditioned group, this ratio was lower and about two times more than Meany (Meaney, 2003) suggested. Our model’s range of axon shear modulus changes generally overlaps with recent research (Table 6). However, there are still differences of opinion in the research (Saeidi et al., 2023; Hoursan et al., 2020). Of course, no micromechanical model has presented the behavior of brain tissue under changes caused by preconditioning. As a result, considering the limitations of previous research, such contradictions are expected. Furthermore, some authors have assumed the ECM to be three times softer than axonal fibers $\left(\mu_{\mathrm{axon}}/\mu_{\mathrm{ECM}}=3\right)$ based on limited experimental results, which do not account for large deformations (Karami et al., 2009; Pan et al., 2013; Arbogast and Margulies, 1997). Our control group had a mentioned ratio of 9.3, and our preconditioned group had a ratio of about 6. This ratio was presented as 7.5 by Hoursan et al. (2020), Yousefsani et al. (2018a), and Meaney (2003) reported approximately 13 and 3, respectively. Our micromechanical models are closest to Horsan et al. ’s model because the histological details are added to the micromechanical model. Previous studies on micromechanical models of the white matter often used simplified methods that lacked or did not use sufficient histological data and ignored the influence of randomized fiber orientations. However, the incorporation of axonal orientation information is critical because studies have shown that computational models basically rely on this information to predict the extent of the damage. The homogeneous responses of our specified RVE models of CR under quasi-static tensile are in good agreement with the experimental data (Figure 10). Our models also show the effect of axon volume fraction in two conditions: before and after preconditioning, corresponding to the mechanical response of white matter structures following experimental findings. This indicates that our micromechanical model is valid with histological information, and the optimized material constants provide reliable estimates of the mechanical behavior of tissue components under quasi-static loading. The random fiber orientations implemented in our study play an important role in the homogenous behavior of white matter structures. By comparing our results with previous statistical models, it is clear that integrating fiber orientation data is essential to capture the mechanical behavior of tissue in quasi-static tension accurately. The local responses of white matter structures to quasi-static tension are significantly influenced by the considered tissue microstructure. The RVE model in the preconditioned group, with a random distribution of axonal fiber positions, diameters, and orientation, results in an oscillatory stress distribution with higher local stresses around lower-diameter axonal fibers. This is also due to the increased orientation of axon fibers due to the preconditioning and rearrangement of the fibers. These observations highlight the importance of employing complex models that account for non-uniform axonal architecture when studying the mechanism of axonal injury. We used the embedded element technique for the micromechanical model and its advantages, such as the fact that the meshes are structurally regular, which facilitates finding pairs of nodes to apply periodic boundary conditions. Also, the independent meshing of the fiber and matrix domain reduces the total number of elements, reducing computational costs compared to the direct mesh method. In this approach, we faced the problem of stiffness redundancy, which was modeled using the appropriate correction method presented in previous studies. The use of the embedded element technique has limitations in presenting the interaction between fibers and matrix, which, of course, was beyond the goals of this research.

Future research should address several limitations identified in this study. More advanced models need to account for the porous nature of brain tissue and the heterogeneous arrangement of axonal fibers across different regions of white matter. Additionally, the extracellular matrix (ECM) should be modeled as a heterogeneous microstructure, incorporating elements like glial cells and capillaries to more accurately reflect tissue complexity. Regarding material parameters, it is important to recognize that parameters calibrated from uniaxial loading conditions do not fully capture the brain’s physiological response during complex *in vivo* deformations. For instance, simulations of slow processes, such as preconditioning, should be based on parameters derived from preconditioned tissue responses, while fast processes should rely on parameters from non-preconditioned tissue, ideally obtained at high strain rates (Budday et al., 2020). Selecting appropriate material parameters is crucial, as simulating strains beyond the calibrated range may lead to significant under- or overestimation of the tissue’s mechanical behavior and injury risk. Given the biological and mechanical complexity of brain tissue, future experimental designs should closely align with the intended clinical applications, and models must be developed with careful consideration of these complexities to improve their translational relevance.

## 7 Conclusion

This study advances our understanding of brain tissue behavior under preconditioning through an integrated approach combining mechanical testing, histological analysis, and micromechanical modeling. We found that preconditioning leads to significant mechanical and microstructural changes in brain tissue, notably affecting axonal integrity. By employing a statistical fiber network model based on histological data, we achieved a more precise representation of the brain’s microstructure. Additionally, our inverse parameter identification process successfully linked these microstructural features to the tissue’s macroscopic mechanical response. The study makes two major contributions. First, we present an experimental study that incorporates histological data to investigate preconditioning effects. Second, we introduce an RVE-based micromechanical modeling framework to further explore these effects. We demonstrate the practicality and effectiveness of using RVE-based homogenization to bridge the gap between microstructural data and macroscopic mechanical behavior. These innovations provide a thorough understanding of brain tissue mechanics under repetitive loading, with far-reaching implications for brain injury research, the development of preconditioning protocols, and future biomechanical modeling efforts.

## Data Availability

The original contributions presented in the study are included in the article/Supplementary Material, further inquiries can be directed to the corresponding author.

## References

[B1] Antonovaite N. Hulshof L. A. Hol E. M. Wadman W. J. Iannuzzi D. (2021). Viscoelastic mapping of mouse brain tissue: relation to structure and age. J. Mech. Behav. Biomed. Mater. 113, 104159. 10.1016/j.jmbbm.2020.104159 33137655

[B2] Arbogast K. B. Margulies S. S. (1997). Regional differences in mechanical properties of the porcine central nervous system. SAE Trans., 3807–3814. 10.4271/973336

[B3] Arbogast K. B. Margulies S. S. (1998). Material characterization of the brainstem from oscillatory shear tests. J. biomechanics 31, 801–807. 10.1016/s0021-9290(98)00068-2 9802780

[B4] Arbogast K. B. Margulies S. S. (1999). A fiber-reinforced composite model of the viscoelastic behavior of the brainstem in shear. J. biomechanics 32, 865–870. 10.1016/s0021-9290(99)00042-1 10433430

[B5] Atashpaz-Gargari E. Lucas C. (2007). “Imperialist competitive algorithm: an algorithm for optimization inspired by imperialistic competition,” in 2007 IEEE congress on evolutionary computation (Ieee), 4661–4667.

[B6] Bankhead P. Loughrey M. B. Fernández J. A. Dombrowski Y. McArt D. G. Dunne P. D. (2017). Qupath: open source software for digital pathology image analysis. Sci. Rep. 7, 16878–16885. 10.1038/s41598-017-17204-5 29203879 PMC5715110

[B7] Barnes J. M. Przybyla L. Weaver V. M. (2017). Tissue mechanics regulate brain development, homeostasis and disease. J. cell Sci. 130, 71–82. 10.1242/jcs.191742 28043968 PMC5394781

[B8] Barros C. S. Franco S. J. Müller U. (2011). Extracellular matrix: functions in the nervous system. Cold Spring Harb. Perspect. Biol. 3, a005108. 10.1101/cshperspect.a005108 21123393 PMC3003458

[B10] Bilston L. E. Liu Z. Phan-Thien N. (2001). Large strain behaviour of brain tissue in shear: some experimental data and differential constitutive model. Biorheology 38, 335–345. 11673648

[B11] Bouaoune L. Brunet Y. El Moumen A. Kanit T. Mazouz H. (2016). Random versus periodic microstructures for elasticity of fibers reinforced composites. Compos. Part B Eng. 103, 68–73. 10.1016/j.compositesb.2016.08.026

[B12] Bruns Jr J. Hauser W. A. (2003). The epidemiology of traumatic brain injury: a review. Epilepsia 44, 2–10. 10.1046/j.1528-1157.44.s10.3.x 14511388

[B13] Budday S. Nay R. de Rooij R. Steinmann P. Wyrobek T. Ovaert T. C. (2015). Mechanical properties of gray and white matter brain tissue by indentation. J. Mech. Behav. Biomed. Mater. 46, 318–330. 10.1016/j.jmbbm.2015.02.024 25819199 PMC4395547

[B14] Budday S. Ovaert T. C. Holzapfel G. A. Steinmann P. Kuhl E. (2020). Fifty shades of brain: a review on the mechanical testing and modeling of brain tissue. Archives Comput. Methods Eng. 27, 1187–1230. 10.1007/s11831-019-09352-w

[B15] Budday S. Raybaud C. Kuhl E. (2014). A mechanical model predicts morphological abnormalities in the developing human brain. Sci. Rep. 4, 5644. 10.1038/srep05644 25008163 PMC4090617

[B16] Budday S. Sommer G. Birkl C. Langkammer C. Haybaeck J. Kohnert J. (2017). Mechanical characterization of human brain tissue. Acta biomater. 48, 319–340. 10.1016/j.actbio.2016.10.036 27989920

[B17] Carew E. Barber J. Vesely I. (2000). Role of preconditioning and recovery time in repeated testing of aortic valve tissues: validation through quasilinear viscoelastic theory. Ann. Biomed. Eng. 28, 1093–1100. 10.1114/1.1310221 11132193

[B18] Carew E. O. Garg A. Barber J. E. Vesely I. (2004). Stress relaxation preconditioning of porcine aortic valves. Ann. Biomed. Eng. 32, 563–572. 10.1023/b:abme.0000019176.49650.19 15117030

[B19] Carriel V. Campos A. Alaminos M. Raimondo S. Geuna S. (2017). Staining methods for normal and regenerative myelin in the nervous system. Histochem. single Mol. methods Protoc. 1560, 207–218. 10.1007/978-1-4939-6788-9_15 28155156

[B20] Chavoshnejad P. German G. K. Razavi M. J. (2021). Hyperelastic material properties of axonal fibers in brain white matter. Brain Multiphysics 2, 100035. 10.1016/j.brain.2021.100035

[B21] Cheng S. Bilston L. E. (2007). Unconfined compression of white matter. J. biomechanics 40, 117–124. 10.1016/j.jbiomech.2005.11.004 16376349

[B22] Cheng S. Clarke E. C. Bilston L. E. (2009). The effects of preconditioning strain on measured tissue properties. J. biomechanics 42, 1360–1362. 10.1016/j.jbiomech.2009.03.023 19394022

[B23] Cloots R. Van Dommelen J. Nyberg T. Kleiven S. Geers M. (2011). Micromechanics of diffuse axonal injury: influence of axonal orientation and anisotropy. Biomechanics Model. Mechanobiol. 10, 413–422. 10.1007/s10237-010-0243-5 20635116

[B24] Dalbosco M. Carniel T. A. Fancello E. A. Holzapfel G. A. (2021). Multiscale numerical analyses of arterial tissue with embedded elements in the finite strain regime. Comput. Methods Appl. Mech. Eng. 381, 113844. 10.1016/j.cma.2021.113844

[B25] Darvish K. Crandall J. (2001). Nonlinear viscoelastic effects in oscillatory shear deformation of brain tissue. Med. Eng. & Phys. 23, 633–645. 10.1016/s1350-4533(01)00101-1 11755808

[B26] Dassault Systèmes (2021). Abaqus analysis user’s manual. 2021 edn. Providence, RI: Dassault Systèmes.

[B27] de Rooij R. Kuhl E. (2016). Constitutive modeling of brain tissue: current perspectives. Appl. Mech. Rev. 68. 10.1115/1.4032436

[B28] De Souza Neto E. A. Blanco P. J. Sánchez P. J. Feijóo R. A. (2015). An rve-based multiscale theory of solids with micro-scale inertia and body force effects. Mech. Mater. 80, 136–144. 10.1016/j.mechmat.2014.10.007

[B29] Destrade M. Gilchrist M. D. Murphy J. G. Rashid B. Saccomandi G. (2015). Extreme softness of brain matter in simple shear. Int. J. Non-Linear Mech. 75, 54–58. 10.1016/j.ijnonlinmec.2015.02.014

[B30] Ehret A. E. Itskov M. (2007). A polyconvex hyperelastic model for fiber-reinforced materials in application to soft tissues. J. Mater. Sci. 42, 8853–8863. 10.1007/s10853-007-1812-6

[B31] Elkin B. S. Ilankovan A. Morrison I. I. I. B. (2010). Age-dependent regional mechanical properties of the rat hippocampus and cortex. J. Biomech. Eng. 132, 011010. 10.1115/1.4000164 20524748

[B32] Elwi A. E. Hrudey T. M. (1991). Closure to “ *finite element Model for curved embedded reinforcement* ” by alaa E. Elwi and terry M. Hrudey (april, 1989, vol. 115, No. 4). J. Eng. Mech. 117, 714–715. (april, 1989, vol. 115, no. 4). 10.1061/(asce)0733-9399(1991)117:3(714.2)

[B33] Eskandari F. Shafieian M. Aghdam M. M. Laksari K. (2021). Tension strain-softening and compression strain-stiffening behavior of brain white matter. Ann. Biomed. Eng. 49, 276–286. 10.1007/s10439-020-02541-w 32494967

[B34] Fawcett J. W. Verhaagen J. (2018). Intrinsic determinants of axon regeneration. Dev. Neurobiol. 78, 890–897. 10.1002/dneu.22637 30345655

[B35] Feng Y. Lee C.-H. Sun L. Ji S. Zhao X. (2017). Characterizing white matter tissue in large strain via asymmetric indentation and inverse finite element modeling. J. Mech. Behav. Biomed. Mater. 65, 490–501. 10.1016/j.jmbbm.2016.09.020 27665084 PMC5154882

[B36] Finan J. D. Elkin B. S. Pearson E. M. Kalbian I. L. Morrison B. (2012). Viscoelastic properties of the rat brain in the sagittal plane: effects of anatomical structure and age. Ann. Biomed. Eng. 40, 70–78. 10.1007/s10439-011-0394-2 22012082

[B37] Franceschini G. Bigoni D. Regitnig P. Holzapfel G. A. (2006). Brain tissue deforms similarly to filled elastomers and follows consolidation theory. J. Mech. Phys. Solids 54, 2592–2620. 10.1016/j.jmps.2006.05.004

[B38] Fung Y. (1993). “Biomechanics: mechanical properties of living tissues,” in Biomechanics. New York: Springer.

[B39] Galford J. E. McElhaney J. H. (1970). A viscoelastic study of scalp, brain, and dura. J. biomechanics 3, 211–221. 10.1016/0021-9290(70)90007-2 5521539

[B40] Garcia-Gonzalez D. Jérusalem A. Garzon-Hernandez S. Zaera R. Arias A. (2018). A continuum mechanics constitutive framework for transverse isotropic soft tissues. J. Mech. Phys. Solids 112, 209–224. 10.1016/j.jmps.2017.12.001

[B41] Garimella H. T. Menghani R. R. Gerber J. I. Sridhar S. Kraft R. H. (2019). Embedded finite elements for modeling axonal injury. Ann. Biomed. Eng. 47, 1889–1907. 10.1007/s10439-018-02166-0 30519759

[B42] Garo A. Hrapko M. Van Dommelen J. Peters G. (2007). Towards a reliable characterisation of the mechanical behaviour of brain tissue: the effects of post-mortem time and sample preparation. Biorheology 44, 51–58. 17502689

[B43] Geers M. G. Kouznetsova V. G. Brekelmans W. (2010). Multi-scale computational homogenization: trends and challenges. J. Comput. Appl. Math. 234, 2175–2182. 10.1016/j.cam.2009.08.077

[B44] Gefen A. Gefen N. Zhu Q. Raghupathi R. Margulies S. S. (2003). Age-dependent changes in material properties of the brain and braincase of the rat. J. neurotrauma 20, 1163–1177. 10.1089/089771503770802853 14651804

[B45] Gefen A. Margulies S. S. (2004). Are *in vivo* and *in situ* brain tissues mechanically similar? J. biomechanics 37, 1339–1352. 10.1016/j.jbiomech.2003.12.032 15275841

[B46] Giordano C. Kleiven S. (2014). Connecting fractional anisotropy from medical images with mechanical anisotropy of a hyperviscoelastic fibre-reinforced constitutive model for brain tissue. J. R. Soc. Interface 11, 20130914. 10.1098/rsif.2013.0914 24258158 PMC3869163

[B47] Goriely A. Geers M. G. Holzapfel G. A. Jayamohan J. Jérusalem A. Sivaloganathan S. (2015). Mechanics of the brain: perspectives, challenges, and opportunities. Biomechanics Model. Mechanobiol. 14, 931–965. 10.1007/s10237-015-0662-4 PMC4562999 25716305

[B48] Goudarzi M. Simone A. (2019). Discrete inclusion models for reinforced composites: comparative performance analysis and modeling challenges. Comput. Methods Appl. Mech. Eng. 355, 535–557. 10.1016/j.cma.2019.06.026

[B49] Hazanov S. Huet C. (1994). Order relationships for boundary conditions effect in heterogeneous bodies smaller than the representative volume. J. Mech. Phys. Solids 42, 1995–2011. 10.1016/0022-5096(94)90022-1

[B50] Hill R. (1963). Elastic properties of reinforced solids: some theoretical principles. J. Mech. Phys. Solids 11, 357–372. 10.1016/0022-5096(63)90036-x

[B51] Hill R. (1965a). Continuum micro-mechanics of elastoplastic polycrystals. J. Mech. Phys. Solids 13, 89–101. 10.1016/0022-5096(65)90023-2

[B52] Hill R. (1965b). A self-consistent mechanics of composite materials. J. Mech. Phys. Solids 13, 213–222. 10.1016/0022-5096(65)90010-4

[B53] Hill R. (1972). On constitutive macro-variables for heterogeneous solids at finite strain. Proc. R. Soc. Lond. A. Math. Phys. Sci. 326, 131–147.

[B54] Holzapfel G. A. (2002). Nonlinear solid mechanics: a continuum approach for engineering science. Meccanica

[B55] Holzapfel G. A. Fereidoonnezhad B. (2017). “Modeling of damage in soft biological tissues,” in Biomechanics of living organs (Elsevier), 101–123.

[B56] Holzapfel G. A. Ogden R. W. (2009). Constitutive modelling of passive myocardium: a structurally based framework for material characterization. Philosophical Trans. R. Soc. A Math. Phys. Eng. Sci. 367, 3445–3475. 10.1098/rsta.2009.0091 19657007

[B57] Hoppstädter M. Püllmann D. Seydewitz R. Kuhl E. Böl M. (2022). Correlating the microstructural architecture and macrostructural behaviour of the brain. Acta Biomater. 151, 379–395. 10.1016/j.actbio.2022.08.034 36002124

[B58] Hori M. Nemat-Nasser S. (1999). On two micromechanics theories for determining micro–macro relations in heterogeneous solids. Mech. Mater. 31, 667–682. 10.1016/s0167-6636(99)00020-4

[B59] Hoursan H. Farahmand F. Ahmadian M. T. (2020). A three-dimensional statistical volume element for histology informed micromechanical modeling of brain white matter. Ann. Biomed. Eng. 48, 1337–1353. 10.1007/s10439-020-02458-4 31965358

[B60] Hrapko M. Van Dommelen J. Peters G. Wismans J. (2008). Characterisation of the mechanical behaviour of brain tissue in compression and shear. Biorheology 45, 663–676. 10.3233/bir-2008-0512 19065013

[B61] Jin X. Zhu F. Mao H. Shen M. Yang K. H. (2013). A comprehensive experimental study on material properties of human brain tissue. J. biomechanics 46, 2795–2801. 10.1016/j.jbiomech.2013.09.001 24112782

[B62] Johnson V. E. Stewart W. Smith D. H. (2013). Axonal pathology in traumatic brain injury. Exp. Neurol. 246, 35–43. 10.1016/j.expneurol.2012.01.013 22285252 PMC3979341

[B63] Karami G. Grundman N. Abolfathi N. Naik A. Ziejewski M. (2009). A micromechanical hyperelastic modeling of brain white matter under large deformation. J. Mech. Behav. Biomed. Mater. 2, 243–254. 10.1016/j.jmbbm.2008.08.003 19627829

[B64] Kaster T. Sack I. Samani A. (2011). Measurement of the hyperelastic properties of *ex vivo* brain tissue slices. J. biomechanics 44, 1158–1163. 10.1016/j.jbiomech.2011.01.019 21329927

[B65] Kolias A. G. Kirkpatrick P. J. Hutchinson P. J. (2013). Decompressive craniectomy: past, present and future. Nat. Rev. Neurol. 9, 405–415. 10.1038/nrneurol.2013.106 23752906

[B66] Koser D. E. Moeendarbary E. Hanne J. Kuerten S. Franze K. (2015). Cns cell distribution and axon orientation determine local spinal cord mechanical properties. Biophysical J. 108, 2137–2147. 10.1016/j.bpj.2015.03.039 PMC4423070 25954872

[B67] Laksari K. Shafieian M. Darvish K. (2012). Constitutive model for brain tissue under finite compression. J. biomechanics 45, 642–646. 10.1016/j.jbiomech.2011.12.023 22281404

[B68] Lee S. King M. Sun J. Xie H. Subhash G. Sarntinoranont M. (2014). Measurement of viscoelastic properties in multiple anatomical regions of acute rat brain tissue slices. J. Mech. Behav. Biomed. Mater. 29, 213–224. 10.1016/j.jmbbm.2013.08.026 24099950 PMC8011428

[B69] Li X. von Holst H. Kleiven S. (2011). Influence of gravity for optimal head positions in the treatment of head injury patients. Acta Neurochir. 153, 2057–2064. 10.1007/s00701-011-1078-2 21739174

[B70] Li Z. Yang H. Wang G. Han X. Zhang S. (2019). Compressive properties and constitutive modeling of different regions of 8-week-old pediatric porcine brain under large strain and wide strain rates. J. Mech. Behav. Biomed. Mater. 89, 122–131. 10.1016/j.jmbbm.2018.09.010 30268868

[B71] Libertiaux V. Pascon F. Cescotto S. (2011). Experimental verification of brain tissue incompressibility using digital image correlation. J. Mech. Behav. Biomed. Mater. 4, 1177–1185. 10.1016/j.jmbbm.2011.03.028 21783126

[B72] MacManus D. B. Menichetti A. Depreitere B. Famaey N. Vander Sloten J. Gilchrist M. (2020). Towards animal surrogates for characterising large strain dynamic mechanical properties of human brain tissue. Brain Multiphysics 1, 100018. 10.1016/j.brain.2020.100018

[B73] MacManus D. B. Murphy J. G. Gilchrist M. D. (2018). Mechanical characterisation of brain tissue up to 35% strain at 1, 10, and 100/s using a custom-built micro-indentation apparatus. J. Mech. Behav. Biomed. Mater. 87, 256–266. 10.1016/j.jmbbm.2018.07.025 30096513

[B74] MacManus D. B. Pierrat B. Murphy J. G. Gilchrist M. D. (2017). Region and species dependent mechanical properties of adolescent and young adult brain tissue. Sci. Rep. 7, 13729. 10.1038/s41598-017-13727-z 29061984 PMC5653834

[B75] Majdan M. Plancikova D. Brazinova A. Rusnak M. Nieboer D. Feigin V. (2016). Epidemiology of traumatic brain injuries in europe: a cross-sectional analysis. Lancet Public Health 1, e76–e83. 10.1016/s2468-2667(16)30017-2 29253420

[B76] Mandel J. (1971). Plasticite classique et viscoplasticite. course and lectures. Int. Centre Mech. Sci. 97.

[B77] Meaney D. F. (2003). Relationship between structural modeling and hyperelastic material behavior: application to cns white matter. Biomechanics Model. Mechanobiol. 1, 279–293. 10.1007/s10237-002-0020-1 14586696

[B78] Menichetti A. MacManus D. B. Gilchrist M. D. Depreitere B. Vander Sloten J. Famaey N. (2020). Regional characterization of the dynamic mechanical properties of human brain tissue by microindentation. Int. J. Eng. Sci. 155, 103355. 10.1016/j.ijengsci.2020.103355

[B79] Metz H. McElhaney J. Ommaya A. K. (1970). A comparison of the elasticity of live, dead, and fixed brain tissue. J. biomechanics 3, 453–458. 10.1016/0021-9290(70)90017-5 5000415

[B80] Mihai L. A. Chin L. Janmey P. A. Goriely A. (2015). A comparison of hyperelastic constitutive models applicable to brain and fat tissues. J. R. Soc. Interface 12, 20150486. 10.1098/rsif.2015.0486 26354826 PMC4614457

[B81] Mihai L. A. Goriely A. (2017). How to characterize a nonlinear elastic material? a review on nonlinear constitutive parameters in isotropic finite elasticity. Proc. R. Soc. A Math. Phys. Eng. Sci. 473, 20170607. 10.1098/rspa.2017.0607 PMC5719638 29225507

[B82] Miller K. Chinzei K. (1997). Constitutive modelling of brain tissue: experiment and theory. J. biomechanics 30, 1115–1121. 10.1016/s0021-9290(97)00092-4 9456379

[B83] Miller K. Chinzei K. (2002). Mechanical properties of brain tissue in tension. J. biomechanics 35, 483–490. 10.1016/s0021-9290(01)00234-2 11934417

[B84] Mrozek S. Vardon F. Geeraerts T. (2012). Brain temperature: physiology and pathophysiology after brain injury. Anesthesiol. Res. Pract. 2012, 1–13. 10.1155/2012/989487 PMC3541556 23326261

[B85] Nicolle S. Lounis M. Willinger R. (2004). “Shear properties of brain tissue over a frequency range relevant for automotive impact situations: new experimental results,” in Tech. rep. (SAE Technical Paper). 10.4271/2004-22-0011 17230269

[B86] Ning X. Zhu Q. Lanir Y. Margulies S. S. (2006). A transversely isotropic viscoelastic constitutive equation for brainstem undergoing finite deformation. J. Biomech. Eng. 128 (6), 925–933. 10.1115/1.2354208 17154695

[B87] Ogden R. W. (1972). Large deformation isotropic elasticity–on the correlation of theory and experiment for incompressible rubberlike solids. Proc. R. Soc. Lond. A. Math. Phys. Sci. 326, 565–584.

[B88] Pan Y. Sullivan D. Shreiber D. I. Pelegri A. A. (2013). Finite element modeling of cns white matter kinematics: use of a 3d rve to determine material properties. Front. Bioeng. Biotechnol. 1, 19. 10.3389/fbioe.2013.00019 25152875 PMC4126384

[B89] Phillips D. Zienkiewicz O. (1976). “Finite element non-linear analysis of concrete structures,” in Proceedings of the Institution of Civil Engineers 61, 59–88. 10.1680/iicep.1976.3503

[B90] Pinto J. Patitucci P. (1980). Visco-elasticity of passive cardiac muscle. J. Biomech. Eng. 102 (1), 57–61. 10.1115/1.3138199 7382454

[B91] Pogoda K. Chin L. Georges P. C. Byfield F. J. Bucki R. Kim R. (2014). Compression stiffening of brain and its effect on mechanosensing by glioma cells. New J. Phys. 16, 075002. 10.1088/1367-2630/16/7/075002 PMC4380293 25844043

[B92] Prange M. T. Margulies S. S. (2002). Regional, directional, and age-dependent properties of the brain undergoing large deformation. J. Biomech. Eng. 124, 244–252. 10.1115/1.1449907 12002135

[B93] Prevost T. P. Balakrishnan A. Suresh S. Socrate S. (2011). Biomechanics of brain tissue. Acta biomater. 7, 83–95. 10.1016/j.actbio.2010.06.035 20603231

[B94] Qian L. Zhao H. Guo Y. Li Y. Zhou M. Yang L. (2018). Influence of strain rate on indentation response of porcine brain. J. Mech. Behav. Biomed. Mater. 82, 210–217. 10.1016/j.jmbbm.2018.03.031 29621688

[B95] Rashid B. Destrade M. Gilchrist M. D. (2012). Mechanical characterization of brain tissue in compression at dynamic strain rates. J. Mech. Behav. Biomed. Mater. 10, 23–38. 10.1016/j.jmbbm.2012.01.022 22520416

[B96] Rashid B. Destrade M. Gilchrist M. D. (2013). Mechanical characterization of brain tissue in simple shear at dynamic strain rates. J. Mech. Behav. Biomed. Mater. 28, 71–85. 10.1016/j.jmbbm.2013.07.017 23973615

[B97] Rashid B. Destrade M. Gilchrist M. D. (2014). Mechanical characterization of brain tissue in tension at dynamic strain rates. J. Mech. Behav. Biomed. Mater. 33, 43–54. 10.1016/j.jmbbm.2012.07.015 23127641

[B98] Reiter N. Schäfer A.-M. Auer S. Paulsen F. Budday S. (2023). Modeling the finite viscoelasticity of human brain tissue based on microstructural information. PAMM 23, e202300234. 10.1002/pamm.202300234

[B99] Rueden C. T. Schindelin J. Hiner M. C. DeZonia B. E. Walter A. E. Arena E. T. (2017). Imagej2: imagej for the next generation of scientific image data. BMC Bioinforma. 18, 529–626. 10.1186/s12859-017-1934-z PMC5708080 29187165

[B100] Saeidi S. Kainz M. P. Dalbosco M. Terzano M. Holzapfel G. A. (2023). Histology-informed multiscale modeling of human brain white matter. Sci. Rep. 13, 19641. 10.1038/s41598-023-46600-3 37949949 PMC10638412

[B101] Samadi-Dooki A. Voyiadjis G. Z. Stout R. W. (2017). An indirect indentation method for evaluating the linear viscoelastic properties of the brain tissue. J. biomechanical Eng. 139, 061007. 10.1115/1.4036486 28418454

[B102] Sargon M. F. Celik H. H. Akşi̇t M. D. Karaağaoğlu E. (2007). Quantitative analysis of myelinated axons of corpus callosum in the human brain. Int. J. Neurosci. 117, 749–755. 10.1080/00207450600910119 17454242

[B103] Sepehrband F. Alexander D. C. Clark K. A. Kurniawan N. D. Yang Z. Reutens D. C. (2016). Parametric probability distribution functions for axon diameters of corpus callosum. Front. Neuroanat. 10, 59. 10.3389/fnana.2016.00059 27303273 PMC4880597

[B104] Shuck L. Advani S. (1972). Rheological response of human brain tissue in shear, J. Basic Eng. 94, 905, 911. 10.1115/1.3425588

[B105] Shulyakov A. V. Cenkowski S. S. Buist R. J. Del Bigio M. R. (2011). Age-dependence of intracranial viscoelastic properties in living rats. J. Mech. Behav. Biomed. Mater. 4, 484–497. 10.1016/j.jmbbm.2010.12.012 21316637

[B106] Smith D. H. Hicks R. Povlishock J. T. (2013). Therapy development for diffuse axonal injury. J. neurotrauma 30, 307–323. 10.1089/neu.2012.2825 23252624 PMC3627407

[B107] Sundaresh S. N. Finan J. D. Elkin B. S. Basilio A. V. McKhann G. M. Morrison I. I. I. B. (2022). Region-dependent viscoelastic properties of human brain tissue under large deformations. Ann. Biomed. Eng. 50, 1452–1460. 10.1007/s10439-022-02910-7 35034227

[B108] Suquet P. (1987). “Elements of homogenization for inelastic solid mechanics,” in Homogenization techniques for composite media.

[B109] Sverdlik A. Lanir Y. (2002). Time-dependent mechanical behavior of sheep digital tendons, including the effects of preconditioning. J. Biomech. Eng. 124, 78–84. 10.1115/1.1427699 11871608

[B110] Takhounts E. G. Crandall J. R. Darvish K. (2003). On the importance of nonlinearity of brain tissue under large deformations. Stapp car crash J. 47, 79–92. 10.4271/2003-22-0005 17096245

[B111] Thibault K. L. Margulies S. S. (1998). Age-dependent material properties of the porcine cerebrum: effect on pediatric inertial head injury criteria. J. biomechanics 31, 1119–1126. 10.1016/s0021-9290(98)00122-5 9882044

[B113] Tyler W. J. (2012). The mechanobiology of brain function. Nat. Rev. Neurosci. 13, 867–878. 10.1038/nrn3383 23165263

[B114] Urbanek F. Frink M. (2012). Current opinions on epidemiology, treatment and outcome after traumatic brain injury. J. Trauma Treat. S 1, 2167–1222. 10.4172/2167-1222.1000S1-001

[B115] Velardi F. Fraternali F. Angelillo M. (2006). Anisotropic constitutive equations and experimental tensile behavior of brain tissue. Biomechanics Model. Mechanobiol. 5, 53–61. 10.1007/s10237-005-0007-9 16315049

[B116] Verkhratsky A. Zorec R. Rodríguez J. J. Parpura V. (2016). Astroglia dynamics in ageing and alzheimer’s disease. Curr. Opin. Pharmacol. 26, 74–79. 10.1016/j.coph.2015.09.011 26515274

[B117] Wang P. Du Z. Shi H. Liu J. Liu Z. Zhuang Z. (2023). Origins of brain tissue elasticity under multiple loading modes by analyzing the microstructure-based models. Biomechanics Model. Mechanobiol. 22, 1239–1252. 10.1007/s10237-023-01714-5 37184689

[B118] Wang R. Sarntinoranont M. (2019). Biphasic analysis of rat brain slices under creep indentation shows nonlinear tension-compression behavior. J. Mech. Behav. Biomed. Mater. 89, 1–8. 10.1016/j.jmbbm.2018.08.043 30236976

[B119] Weickenmeier J. de Rooij R. Budday S. Steinmann P. Ovaert T. C. Kuhl E. (2016). Brain stiffness increases with myelin content. Acta biomater. 42, 265–272. 10.1016/j.actbio.2016.07.040 27475531

[B120] Woo S. (1982). Mechanical properties of tendons and ligaments. i. quasi-static and nonlinear viscoelastic properties. Biorheology 19, 385–396. 10.3233/bir-1982-19301 7104480

[B121] Yousefsani S. A. Farahmand F. Shamloo A. (2018a). A three-dimensional micromechanical model of brain white matter with histology-informed probabilistic distribution of axonal fibers. J. Mech. Behav. Biomed. Mater. 88, 288–295. 10.1016/j.jmbbm.2018.08.042 30196184

[B122] Yousefsani S. A. Shamloo A. Farahmand F. (2018b). Micromechanics of brain white matter tissue: a fiber-reinforced hyperelastic model using embedded element technique. J. Mech. Behav. Biomed. Mater. 80, 194–202. 10.1016/j.jmbbm.2018.02.002 29428702

[B123] Yousefsani S. A. Shamloo A. Farahmand F. (2020). Nonlinear mechanics of soft composites: hyperelastic characterization of white matter tissue components. Biomechanics Model. Mechanobiol. 19, 1143–1153. 10.1007/s10237-019-01275-6 31853724

