# Supplementary material for: Toward understanding the brain tissue behavior due to preconditioning: an experimental study and RVE approach
Source: Front Bioeng Biotechnol. 2024 Oct 8;12:1462148. doi: 10.3389/fbioe.2024.1462148 (PMC11493751; doi:10.3389/fbioe.2024.1462148)
Supplement: Supplementary file 1 [file DataSheet1.pdf]

Toward Understanding the Brain Tissue Behavior Due to Preconditioning: An Experimental Study and RVE Approach

Ava Mazhari [1], **Mehdi Shafieian** [1,*]

[1] *Department of Biomedical Engineering, Amirkabir University of Technology (Tehran Polytechnique), Tehran, Iran*

Correspondence*:
Corresponding Author
shafieian@aut.ac.ir

APPENDIX

1 FINITE ELEMENT IMPLEMENTATION OF THE EMBEDDED ELEMENTS TECHNIQUE

To effectively implement a solution for the principle of virtual work within the context of the embedded elements technique (Eq. (**??**)) in the finite strain regime, we employ the finite element method (FEM) within a total Lagrangian framework. For clarity, we consider the specific scenario in which the body $\boldsymbol{\Omega_0}$ deforms into $\boldsymbol{\Omega}$ exclusively due to prescribed displacements $\bar{\mathbf{U}}$, without the influence of body forces $\bar{\mathbf{B}}$ or surface forces $\bar{\mathbf{T}}$. As a result, Eq. (**??**) reduces as follows (considering $\delta\mathbf{F} = \mathrm{Grad}\,\delta\mathbf{u}$):

$$\int_{\Omega_0} \mathbf{P}_m : \delta\mathbf{F}\, dV + \sum_{i=1}^{N_{f_{Total}}} \int_{V_{f_i^0}} \mathbf{P}_{ci} : \delta\mathbf{F}\, dV = 0 \tag{A.1}$$

By subdividing the body Ω_0 into N_{el} continuum finite elements, the principle of virtual work (Eq. (A.1)) can be reformulated as:

$$\mathop{\boldsymbol{A}}_{e=1}^{N_{el}} \left[\int_{\Omega_0^{(e)}} \mathbf{P}_m : \delta\mathbf{F}\, dV + \sum_{i=1}^{N_{f(e)}} \int_{V_0{}^{f_i(e)}} \mathbf{P}_{ci} : \delta\mathbf{F}\, dV \right] = 0 \tag{A.2}$$

Here, $\Omega_0^{(e)}$ represents the initial domain of the eth host element, $\boldsymbol{A}$ is an appropriate assembly operator, and $N_f^{(e)}$ denotes the number of fiber segments entirely contained within the eth host element. It is assumed that each embedded fiber f_i can be divided into smaller segments such that:

$$V_0{}^{f_{Total}} = \sum_{e=1}^{N_{\mathrm{el}}} V_0{}^{f_{Total}(e)} = \sum_{e=1}^{N_{\mathrm{el}}} \sum_{i=1}^{N_f(e)} \int_{V_0{}^{f_i(e)}} dV \tag{A.3}$$

Where ${V_0}^{f_{Total}(e)} \subseteq \Omega_0^{(e)}$ represents the total initial fiber volume within the eth host element, and ${V_0}^{f_{Total}}$ is the total fiber volume embedded in the matrix substance m. By establishing the coordinate system $\{\mathbf{e}_1, \mathbf{e}_2, \mathbf{e}_3\}$, Eq. (A.2) can be expressed in matrix form as:

$$\underset{e=1}{\overset{N_{el}}{\boldsymbol{A}}} \left[\int_{\Omega_0^{(e)}} [\delta \mathbf{F}^{(e)}]^T [\mathbf{P}_m^{(e)}] dV + \sum_{i=1}^{N_f(e)} \int_{{V_0}^{f_i(e)}} [\delta \mathbf{F}^{(e)}]^T [\mathbf{P}_{ci}^{(e)}] dV \right] = 0 \tag{A.4}$$

To approximate $[\delta \mathbf{F}^{(e)}]$, we recall that $\delta \mathbf{F} = \text{Grad}\, \delta \mathbf{u}$ and approximate the field of virtual displacements $\delta \mathbf{u}$ within the eth host element $\Omega_0^{(e)}$ using appropriate shape functions defined in terms of its nodes. After standard manipulations, we obtain:

$$[\mathbf{f}_{\text{int}}] = \underset{e=1}{\overset{N_{el}}{\boldsymbol{A}}} [\mathbf{f}_{\text{int}}^{(e)}] = \underset{e=1}{\overset{N_{el}}{\boldsymbol{A}}} \left[\int_{\Omega_0^{(e)}} [\mathbf{B}^{(e)}]^T [\mathbf{P}_m^{(e)}] dV + \sum_{i=1}^{N_f^{(e)}} \int_{{V_0}^{f_i(e)}} [\mathbf{B}^{(e)}]^T [\mathbf{P}_{ci}^{(e)}] dV \right] \approx [\mathbf{0}] \tag{A.5}$$

Where $[\mathbf{f}_{\text{int}}]$ is the global internal force vector and $[\mathbf{B}^{(e)}]$ is the matrix of derivatives of the shape functions of the eth host element $\Omega_0^{(e)}$. For more details on the FEM derivation, refer to Belytschko et al. (2014).

2 NUMERICAL IMPLEMENTATION OF PERIODIC BOUNDARY CONDITION

In this work, the preprocessing (including the application of periodic boundary conditions), finite element (FE) analysis, and postprocessing involved in evaluating the mechanical properties of composites using the RVE-based FE homogenization method are implemented within the commercial FE software ABAQUS. In ABAQUS, periodic boundary conditions can be enforced on the surface nodes of the composite's representative volume element (RVE) through the use of linear multi-point constraints. This method requires that a linear combination of the nodal displacements satisfy the equation:

$$c_1 u_i^P + c_2 u_j^Q + \cdots + c_N u_k^R = 0, \tag{B.1}$$

where u_i^P is the nodal displacement or a displacement-like field (such as temperature) at the degree of freedom (DOF) i of node P, and c_N are the coefficients defining the relative motion of the nodes.

The displacement constraint described by Equation (1) is introduced into the system of linear multi-point constraint equations by defining a reference point, which is independent of the RVE but provides the required degrees of freedom to control the mechanical response of the RVE. The complete system of equations for the periodic boundary condition can be written as:

$$u_i^+ - u_i^- = u_i^{RP}, \tag{B.2}$$

where the superscripts $+$ and $-$ refer to the corresponding periodic node sets on opposite parallel surfaces of the RVE, and u_i^{RP} is the perturbation at the reference point. This formulation is applied to all relevant nodes on the parallel surfaces of the RVE. It is important to note that once a DOF is included in a constraint equation, it cannot be used in another, as the DOF is eliminated by the constraint. To avoid

over-constraining the system, the constraint equations applied at the edges and vertices of the RVE must be reduced accordingly. To impose periodic boundary conditions on the boundaries of the RVE for composites, the first step is to select and categorize the nodes on the RVE boundaries that need to be constrained. The planar notation and numbering of a cubic RVE are shown in Fig.A1, and the boundary nodes are categorized into three sets:

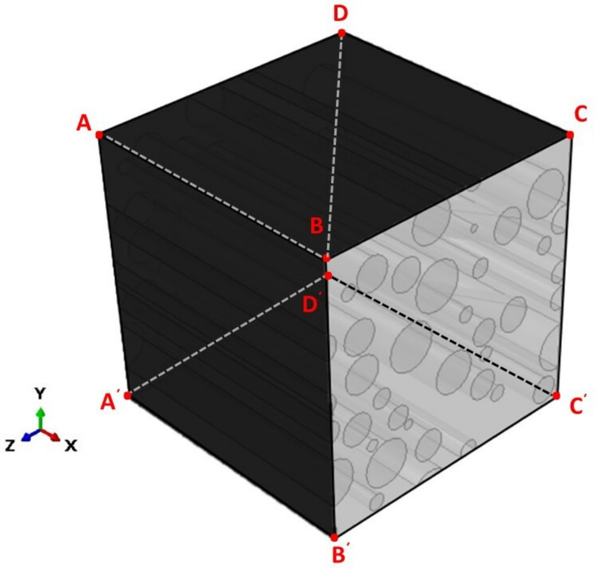

Figure A1. Categorization of node sets on the surfaces of a cubic RVE.

- **Set Inner face:** Includes the bottom face (A'B'C'D'), top face (ABCD), front face (AA'B'B), rear face (DD'C'C), left face (BB'C'C), and right face (AA'D'D).
- **Set Inner edge:** Comprises the edges AA', BB', CC', DD', CD, AB, A'B', C'D', AD, BC, B'C', and A'D'.
- **Set Corner:** Consists of the vertices A, B, C, D, A', B', C', and D'.

Nodes in the *Set Inner face* are those that belong to a single face, excluding nodes on the edges and corners. Nodes in the *Set Inner edge* belong to the edges, excluding the end nodes, which correspond to the corners. Finally, nodes in the *Set Corner* are located at the eight corners of the RVE.

Each loading case specifies displacement fields that nullify all but one of the six independent components of the strain tensor.The detailed implementation of periodic boundary conditions is as follows:

Set I: Inner face

1. Top and Bottom Faces:

$$u_1^{Top} - u_1^{Bottom} - u_1^{Rp-1} = 0, \quad u_2^{Top} - u_2^{Bottom} - u_2^{Rp-3} = 0, \quad u_3^{Top} - u_3^{Bottom} = u_3^{Rp-2} = 0. \quad \text{(B.3)}$$

2. Right and Left Faces:

$$u_1^{Right} - u_1^{Left} - u_1^{Rp-3} = 0, \quad u_2^{Right} - u_2^{Left} - u_2^{Rp-2} = 0, \quad u_3^{Right} - u_3^{Left} - u_3^{Rp-1} = 0. \quad \text{(B.4)}$$

3. Front and Rear Faces:

$$u_1^{Front} - u_1^{Rear} - u_1^{Rp-2} = 0, \quad u_2^{Front} - u_2^{Rear} - u_2^{Rp-1} = 0, \quad u_3^{Front} - u_3^{Rear} - u_3^{Rp-3} = 0. \quad \text{(B.5)}$$

Set II: Inner edge

For the edges AA', BB', CC', and DD', the following conditions hold:

$$u_1^{BB'} - u_1^{AA'} - u_1^{Rp-3} = 0, \quad u_2^{BB'} - u_2^{AA'} - u_2^{Rp-2} = 0, \quad u_3^{BB'} - u_3^{AA'} - u_3^{Rp-1} = 0. \quad \text{(B.6)}$$

$$u_1^{AA'} - u_1^{DD'} - u_1^{Rp-2} = 0, \quad u_2^{AA'} - u_2^{DD'} - u_2^{Rp-1} = 0, \quad u_3^{AA'} - u_3^{DD'} - u_3^{Rp-3} = 0. \quad \text{(B.7)}$$

$$u_1^{CC'} - u_1^{DD'} - u_1^{Rp-3} = 0, \quad u_2^{CC'} - u_2^{DD'} - u_2^{Rp-2} = 0, \quad u_3^{CC'} - u_3^{DD'} - u_3^{Rp-1} = 0. \quad \text{(B.8)}$$

For other edges, similar constraints are applied (details in Tian et al. (2019)).

Set III: Corner

The constraints at vertices A, B, C, and D are expressed as:

$$u_1^{A} - u_1^{D} - u_1^{Rp-2} = 0, \quad u_2^{A} - u_2^{D} - u_2^{Rp-1} = 0, \quad u_3^{A} - u_3^{D} - u_3^{Rp-3} = 0. \quad \text{(B.9)}$$

$$u_1^{C} - u_1^{D} - u_1^{Rp-3} = 0, \quad u_2^{C} - u_2^{D} - u_2^{Rp-2} = 0, \quad u_3^{C} - u_3^{D} - u_3^{Rp-1} = 0. \quad \text{(B.10)}$$

$$u_1^{B} - u_1^{C} - u_1^{Rp-2} = 0, \quad u_2^{B} - u_2^{C} - u_2^{Rp-1} = 0, \quad u_3^{B} - u_3^{C} - u_3^{Rp-3} = 0. \quad \text{(B.11)}$$

Similar constraints are applied for other corners (Tian et al. (2019)). To apply tensile loading, the magnitudes of u_i^{Rp} are chosen accordingly. For tensile loading along the x direction:

$$u_1^{Rp-3} = \bar{\varepsilon}_{11}, \quad u_2^{Rp-3} = 0, \quad u_3^{Rp-3} = 0, u_i^{Rp-1} = 0, \quad u_i^{Rp-2} = 0 \quad \text{(B.12)}$$

(With i = 1,2 and 3 in equation above). This approach ensures that the appropriate loading conditions are applied while maintaining periodic boundary conditions on the RVE.

REFERENCES

Belytschko, T., Liu, W. K., Moran, B., and Elkhodary, K. (2014). *Nonlinear finite elements for continua and structures* (John wiley & sons)

Tian, W., Qi, L., Chao, X., Liang, J., and Fu, M. (2019). Periodic boundary condition and its numerical implementation algorithm for the evaluation of effective mechanical properties of the composites with complicated micro-structures. *Composites Part B: Engineering* 162, 1–10
